# Mitochondrial function meets oncology: the multifaceted role of TFAM across cancer types

**DOI:** 10.1007/s10495-026-02305-2

**Published:** 2026-03-03

**Authors:** Jie Wang, Ruicheng Wu, Fanglin Shao, Zhouting Tuo, Xinrui Li, Koo Han Yoo, Wuran Wei, Zhipeng Wang, Dengxiong Li, Dechao Feng

**Affiliations:** 1 Department of Urology, Urology and Nephrology Center, Zhejiang Provincial People’s Hospital (Affiliated People’s Hospital), Hangzhou Medical College, Hangzhou, 310014 Zhejiang China; 2 Department of Urology, Institute of Urology, West China Hospital, Sichuan University, Chengdu, 610041 China; 3 Department of Urology, Tianjin Institute of Urology, The Second Hospital of Tianjin Medical University, Tianjin, China; 4 Department of Rehabilitation, The Affiliated Hospital of Southwest Medical University, Luzhou, 646000 China; 5 Department of Urology, Kyung Hee University, Seoul, South Korea; 6 Department of Urology, Sichuan Provincial People’s Hospital, University of Electronic Science and Technology of China, Chengdu, 610072 China; 7 Department of Urology, The First Affiliated Hospital of Zhejiang Chinese Medical University (Zhejiang Provincial Hospital of Chinese Medicine), Hangzhou, Zhejiang Province China; 8 Division of Surgery and Interventional Science, University College London, London, W1W 7TS UK

**Keywords:** TFAM, Pan-cancer analysis, Mitochondrial function, Therapeutic strategy, Bioinformatics

## Abstract

Mitochondrial transcription factor A (TFAM) is indispensable for mitochondrial DNA (mtDNA) maintenance and transcription, governing cellular bioenergetics. Despite its known physiological importance, TFAM plays a complex and often paradoxical role in cancer biology. This study integrates pan-cancer bioinformatics analyses with experimental evidence to comprehensively elucidate TFAM’s multifaceted impact on tumorigenesis. We systematically investigated the heterogeneity of TFAM across diverse cancer types, specifically focusing on its regulatory mechanisms in metabolic reprogramming, signal transduction, and immune microenvironment remodeling. Our analysis reveals that TFAM functions as a critical node connecting mitochondrial integrity to tumor progression, balancing tumor-promoting and tumor-suppressive roles depending on the context. Finally, we discuss the challenges of targeting TFAM, such as off-target toxicity, and highlight emerging precision oncology strategies, including mitochondria-targeted delivery systems, that aim to exploit these mitochondrial vulnerabilities.

**Graphical abstract:**

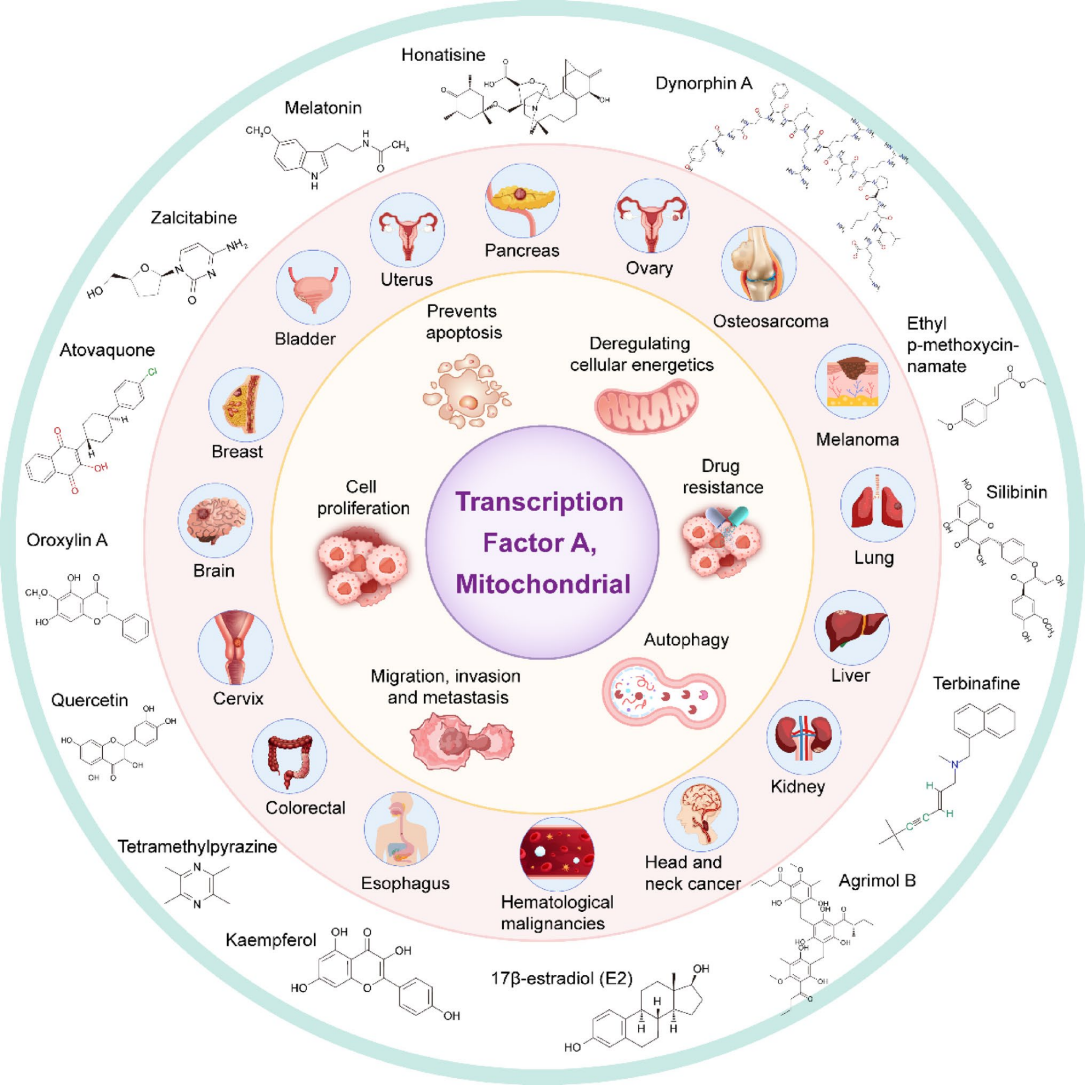

## Introduction

The Transcription Factor A, Mitochondrial (TFAM) gene is located at 10q21.1 and encodes a protein consisting of 246 amino acids with a molecular weight of 29,097 Da. Specifically, TFAM is synthesized in the cytosol and contains an N-terminal mitochondrial targeting sequence spanning residues 1–42. Upon import into the mitochondrial matrix, this signaling sequence is cleaved, resulting in a mature protein of 204 amino acid residues [1]. The mitochondrial transcription initiation complex comprises at least mitochondrial transcription factor B2, TFAM and POLRMT, which are essential for the fundamental transcription of mitochondrial DNA (mtDNA) [2]. Within this complex, TFAM recruits POLRMT to specific promoters, while mitochondrial transcription factor B2 induces structural changes in POLRMT, facilitating the opening of the promoter and the capture of non-template strands of DNA [3]. The expression level of TFAM has been correlated with mtDNA content and gene expression [4]. Furthermore, TFAM plays a crucial role in initiating the replication of mtDNA [5]. In addition to initiating mtDNA transcription and replication, TFAM also maintains the structural integrity of mtDNA [6]. Notably, the role of TFAM in mitochondria resembles that of histones in nucleosomes. TFAM completely wraps mtDNA to form a nucleoid structure [7], which serves to protect mtDNA from reactive oxygen species (ROS) [8].

Mitochondria are organelles that perform oxidative phosphorylation (OXPHOS) to generate the energy required by eukaryotic cells [9, 10]. Additionally, they participate in numerous cellular processes, including signal transduction [11], where they function as dynamic signal-transducing organelles that integrate metabolic and stress cues and communicate with other cellular compartments to shape cellular responses [12], ion homeostasis [13], apoptosis [14], and senescence [15]. Mitochondrial dysfunction has been linked to various pathological phenotypes in humans [16]. Tissue-specific ablation of TFAM has been employed to model the mitochondrial dysfunction observed in several human diseases [17, 18].

Cancer incidence and development are influenced by TFAM [19, 20]. A search of the Web of Science and PubMed databases served as the foundation for the current review. In addition to summary of current publications, we also provide a thorough investigation of the role of TFAM in human cancer through bioinformatics analysis to broaden and deepen our understanding of this gene.

## Regulation of TFAM expression and activity

The expression and function of TFAM are tightly controlled at multiple levels, including transcriptional regulation, post-transcriptional modulation by non-coding RNAs and post-translational modifications, often in response to cellular stress signals.

### Transcriptional control by key signaling pathways

The transcription of the nuclear-encoded *TFAM* gene is primarily regulated by the PGC-1α/NRF axis, a master regulator of mitochondrial biogenesis. PGC-1α co-activates NRF1 and NRF2, which bind to the *TFAM* promoter to drive its expression [21, 22]. This pathway is itself sensitive to cellular energy status and stress. For instance, nutrient deprivation can suppress HIF-1α, leading to increased c-MYC activity, which in turn upregulates NRF1 and TFAM to enhance OXPHOS as an adaptive response [23]. Moreover, several transcription factors have been shown to upregulate TFAM expression in various malignancies, thereby modulating mitochondrial function and contributing to tumor progression: p53 increases mtDNA copy number via TFAM upregulation in colorectal cancer [24]; FoxM1 transcriptionally activates TFAM and regulates mitochondrial dynamics to promote glioblastoma progression [25]; ZNF468 enhances TFAM expression to drive breast cancer growth and cisplatin resistance [26]; NFκB2 coordinates mitochondrial-nuclear genome communication through TFAM regulation in acute myeloid leukemia [27] and POLR1A upregulates TFAM via ATF4 to suppress ferroptosis [28]. Conversely, downregulation of TFAM has been observed through: KLF16, which directly represses TFAM to inhibit glioma cell proliferation [29]; ZNF281, which suppresses the NRF1/PGC-1α-TFAM axis to restrain mitochondrial biogenesis and promote hepatocellular carcinoma metastasis [30] and FBP2, which inhibits NRF1 and TFAM expression to limit mitochondrial biogenesis in sarcoma [31]. Furthermore, oncogenic pathways can hijack this regulation. MARCHF1 promotes breast cancer by facilitating the ubiquitination and degradation of the transcriptional repressor REST, thereby derepressing *TFAM* transcription [32].

### Post-transcriptional regulation by non-coding RNAs

**Fig. 1:**
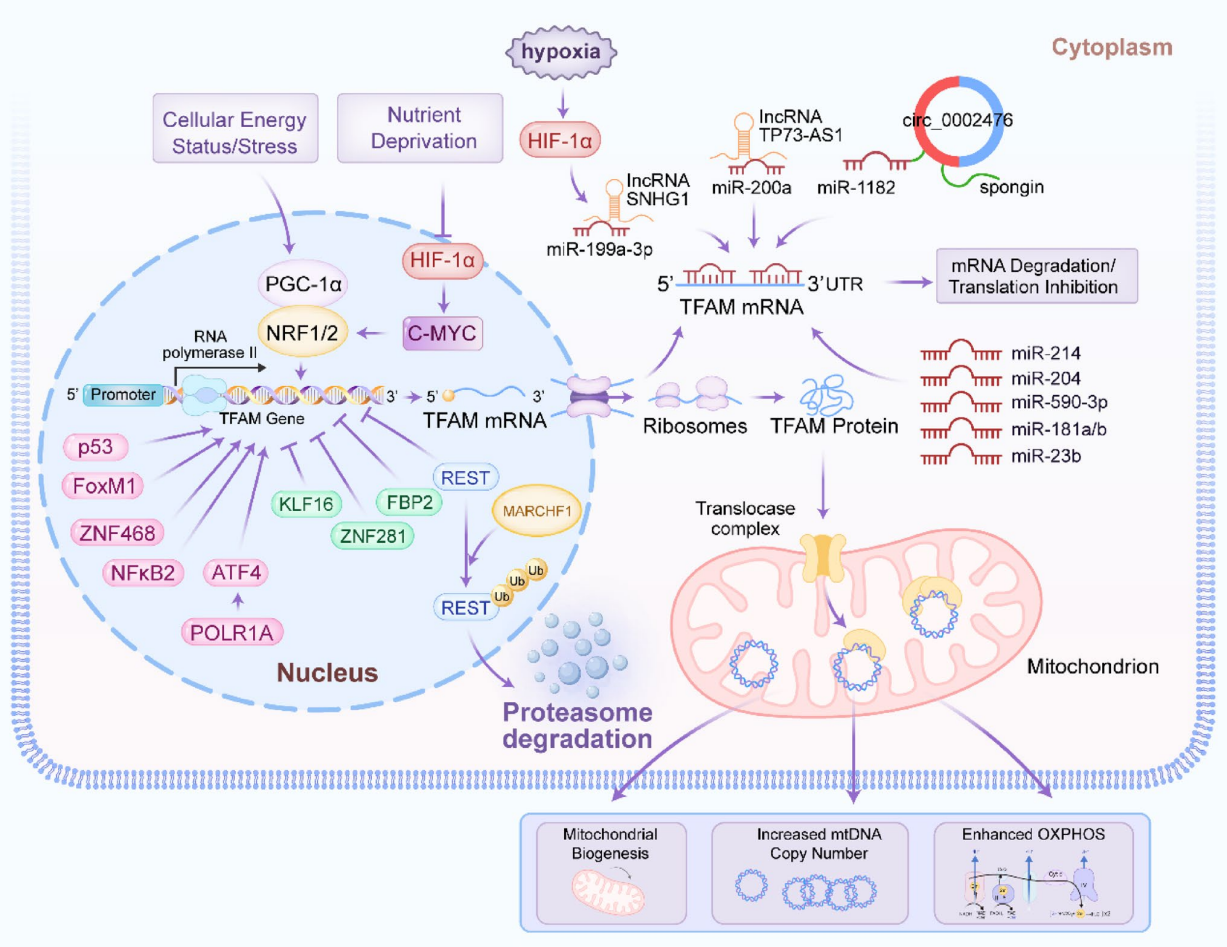
The transcriptional and post-transcriptional regulation of TFAM expression. In the nucleus, cellular energy status/stress activates the PGC-1α-NRF1/2 axis to promote transcription of the *TFAM* gene, whereas microenvironmental cues such as hypoxia and nutrient deprivation engage HIF-1α and c-MYC-associated programs. Multiple additional transcriptional regulators reported in cancer (including p53, FoxM1, ZNF468, NFκB2, ATF4, KLF16, ZNF281, FBP2 and REST) converge on the *TFAM* promoter to fine-tune TFAM mRNA output. In the cytoplasm, TFAM mRNA is targeted by miRNAs (miR-214, miR-204, miR-590-3p, miR-181a/b and miR-23b), leading to mRNA degradation and/or translational repression, while ceRNA networks involving lncRNAs (SNHG1 and TP73-AS1) and a circRNA (circ_0002476) sponge TFAM-targeting miRNAs (e.g., miR-199a-3p, miR-200a and miR-1182) to modulate TFAM translation. lncRNA long non-coding RNA, circRNA circular RNA, miRNA microRNA, ceRNA competing endogenous RNA

Non-coding RNAs (ncRNAs), including microRNAs (miRNAs), long non-coding RNAs (lncRNAs) and circular RNAs (circRNAs), are pivotal post-transcriptional regulators in cancer [33–35]. They control oncogenic processes like proliferation and metastasis by degrading mRNA, inhibiting translation, or sequestering regulatory molecules, thereby modulating key cancer pathways [36–38]. A dense network of miRNAs and lncRNAs fine-tunes TFAM expression, often exhibiting dysregulation in cancer. Multiple miRNAs, including miR-200a, miR-214, miR-204, miR-590-3p, miR-1182, miR-199a-3p, miR-23b and miR-181a/b, have been identified as direct negative regulators of TFAM by binding to its 3′ untranslated region [39–46]. Downregulation of these tumor-suppressive miRNAs (e.g., miR-200a, miR-214, miR-204) in cancers such as breast, colorectal, cervical and bladder leads to TFAM overexpression, driving proliferation and metastasis [39, 41, 47, 48]. Conversely, the lncRNA TP73-AS1 acts as a competing endogenous RNA (ceRNA) in breast cancer, sponging miR-200a to prevent its interaction with *TFAM* mRNA, thereby promoting TFAM expression and proliferation [49]. Similarly, circ_0002476 in non-small cell lung cancer sponges miR-1182 to elevate TFAM levels [43]. Under hypoxic conditions, the lncRNA SNHG1 is induced by HIF-1α and functions as a ceRNA for miR-199a-3p, leading to TFAM upregulation and promoting angiogenesis and metastasis in breast cancer [50]. Figure 1 depicted the transcriptional and post-transcriptional regulation of TFAM.

### Post-translational modifications and protein stability

Beyond transcriptional regulation, the activity and abundance of TFAM are finely tuned by Post-translational modifications and targeted degradation pathways. These mechanisms allow rapid adaptation to tumor microenvironmental stresses, metabolic demands and therapeutic insults, contributing to TFAM’s context-dependent roles in cancer progression and therapy resistance.

TFAM protein stability is primarily governed by two degradation systems: the ubiquitin–proteasome pathway and mitochondrial proteolysis. The E3 ubiquitin ligase Trim21 targets TFAM for ubiquitination and proteasomal degradation. In colorectal cancer, the membrane protein SNAP23 sequesters Trim21 away from mitochondria, reducing TFAM ubiquitination and promoting its accumulation [51]. Elevated TFAM thereby enhances OXPHOS and mitochondrial ROS production, paradoxically sensitizing cells to oxaliplatin by amplifying oxidative stress. Conversely, SNAP23 deficiency releases Trim21, accelerating TFAM turnover and conferring chemoresistance. Within mitochondria, the AAA + protease LONP1 directly degrades TFAM, particularly unbound or damaged forms. In bladder cancer, the cholesterol biosynthesis enzyme SQLE localizes to mitochondria and inhibits LONP1’s proteolytic activity toward TFAM via protein–protein interaction, stabilizing TFAM and boosting OXPHOS to drive tumorigenesis. Disruption of this axis with SQLE inhibitors (e.g., terbinafine) restores TFAM degradation and suppresses growth [52]. Similarly, in pancreatic cancer, miriplatin-loaded liposomes enhance LONP1-mediated degradation of TFAM and POLG, depleting mtDNA and inducing mitophagy, overcoming gemcitabine resistance [53].

Reversible PTMs further modulate TFAM function. Phosphorylation by PKA at residues in the HMG-box domain impairs TFAM’s DNA-binding and transcriptional activation, rendering it susceptible to LONP1 degradation. In colorectal cancer, mitochondrial calcium uptake activates PDE2, suppressing PKA and stabilizing non-phosphorylated TFAM to promote biogenesis and growth [54].

Deacetylation by SIRT3 enhances TFAM’s mtDNA-binding affinity and transcriptional activity. In triple-negative breast cancer, the epigenetic regulator MBD2c recruits SIRT3 to deacetylate TFAM (e.g., at K145/K146), sustaining mtDNA expression and OXPHOS even under cisplatin stress, fostering resistance. This cross-compartmental nuclear-mitochondrial signaling links epigenetic alterations to metabolic resilience [55].

**Fig. 2:**
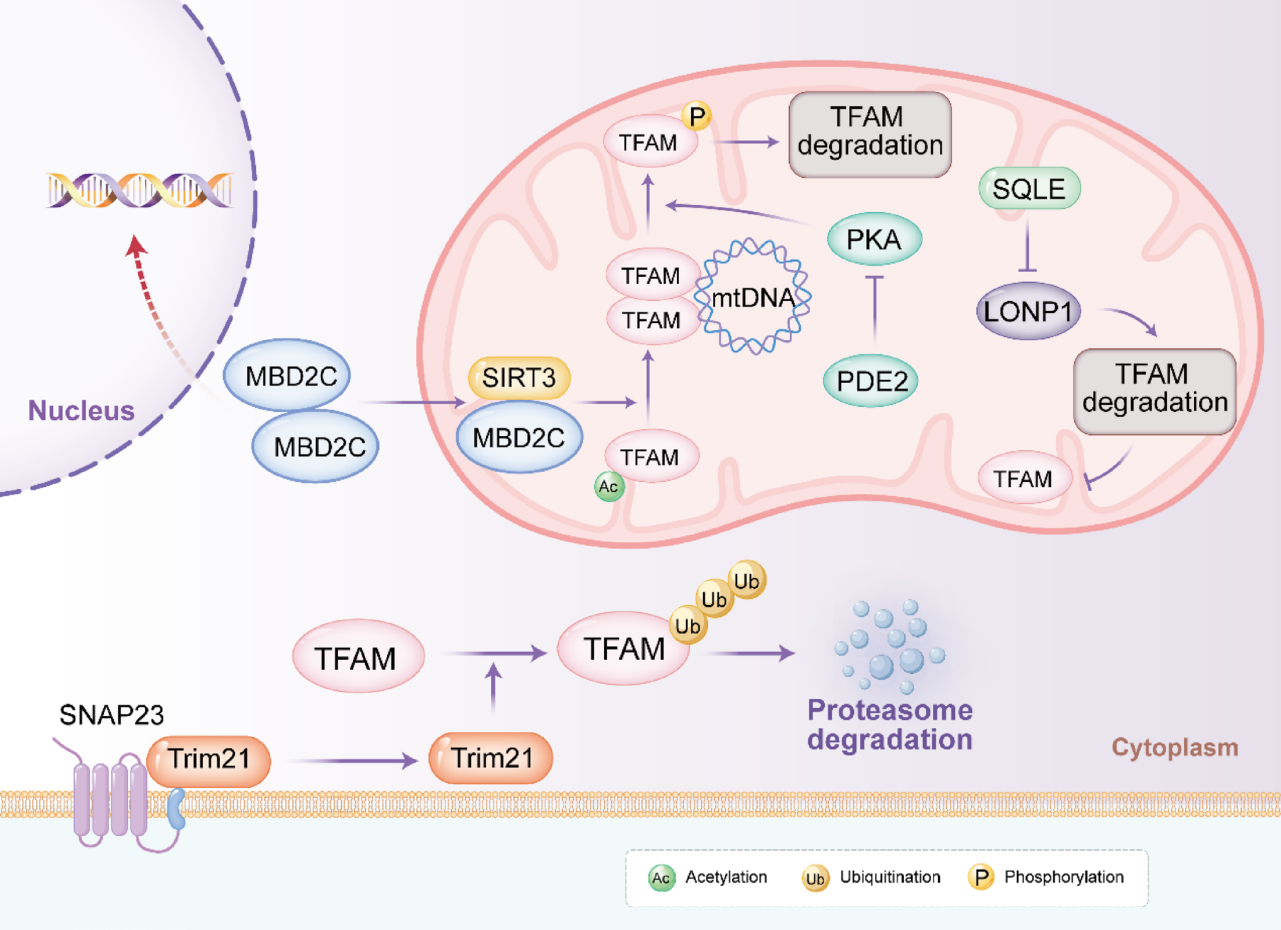
The post-translational modifications of TFAM. In mitochondria, MBD2C cooperates with the mitochondrial deacetylase SIRT3 to modulate the acetylation status of TFAM, thereby influencing TFAM accumulation and its association with mtDNA. TFAM can also undergo phosphorylation, which is linked to reduced TFAM stability and promotes TFAM degradation. This phosphorylation-dependent route is regulated by the PDE2-PKA signaling axis, where PDE2 negatively regulates PKA activity. In addition, mitochondrial proteolysis contributes to TFAM turnover through the SQLE-LONP1 axis, with LONP1 driving TFAM degradation. In the cytoplasm, SNAP23-associated TRIM21 mediates TFAM ubiquitination and targets TFAM for proteasome-dependent degradation. mtDNA mitochondrial DNA

These PTMs and stability controls highlight TFAM’s dynamic regulation in oncology. In OXPHOS-dependent tumors, stabilizing modifications (e.g., deacetylation, reduced ubiquitination/proteolysis) support bioenergetic demands and resistance, while targeted degradation or inhibitory phosphorylation sensitizes cells. Exploiting these pathways via LONP1 activators, SQLE/Trim21 modulators, or SIRT3/PKA interventions, offers promising avenues for precision therapies tailored to mitochondrial vulnerabilities across cancer types. Figure 2 depicted the post-translational modifications of TFAM.

## Pan-cancer RNA-sequencing analysis of TFAM

**Fig. 3:**
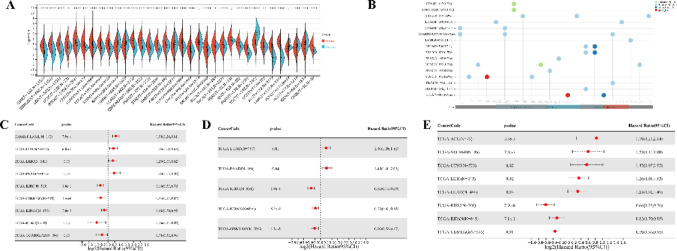
Differential expression and prognosis analyses of TFAM at pan-cancer level. **A** TFAM mRNA expression between tumor and normal tissues at pan-cancer level; **B** mutation landscapes of TFAM at pan-cancer level; **C** pan-cancer analysis of TFAM for overall survival; **D** pan-cancer analysis of TFAM for disease-specific survival; **E** pan-cancer analysis of TFAM for progression-free interval. *p < 0.05; **p < 0.01; ****p < 0.0001

**Fig. 4:**
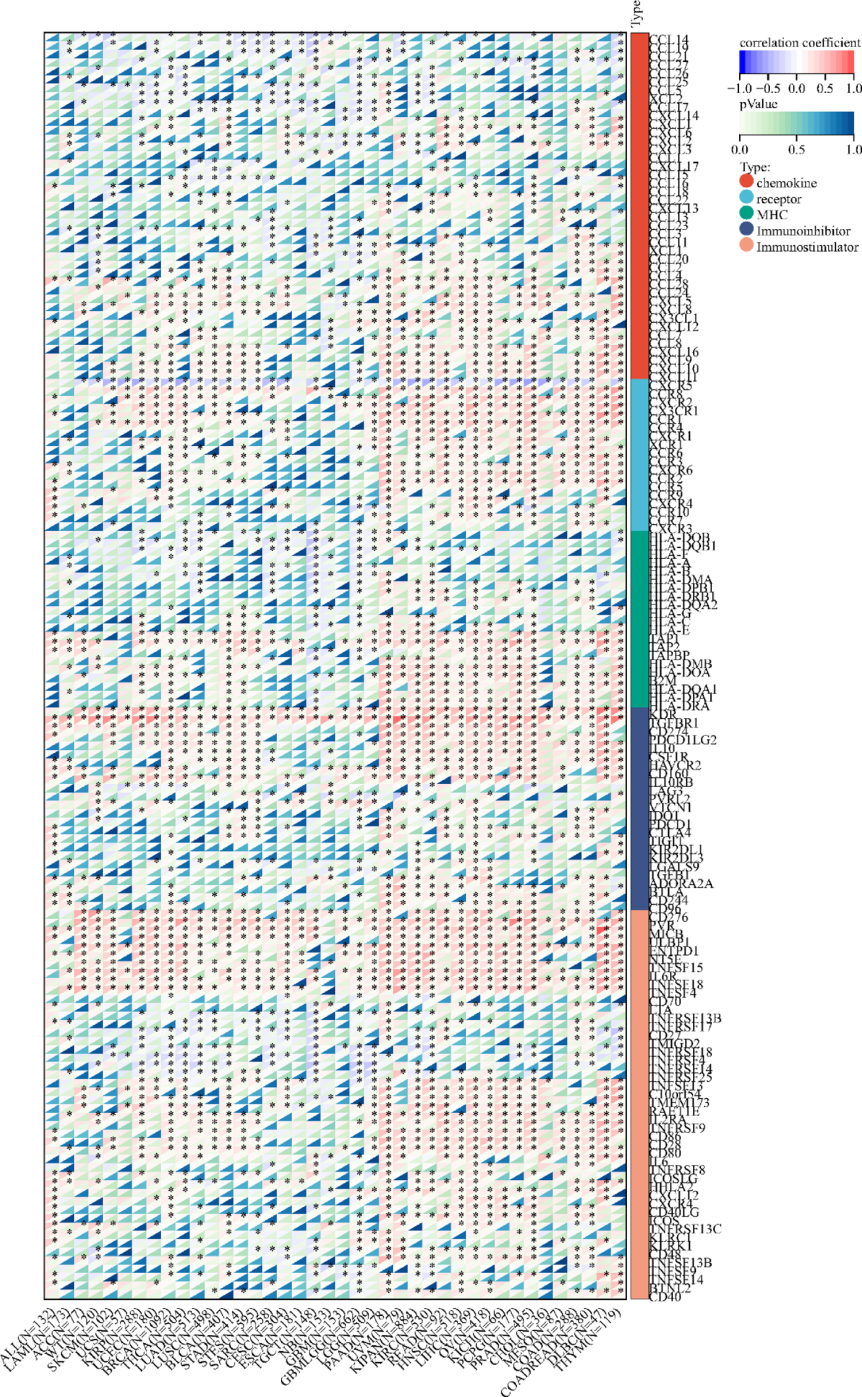
The correlation between TFAM expression and 150 immunomodulators, including chemokines [41], receptors [18], MHC [21], immunoinhibitors [24] and immunostimulators (46) at pan-cancer level. *p < 0.05

**Fig. 5:**
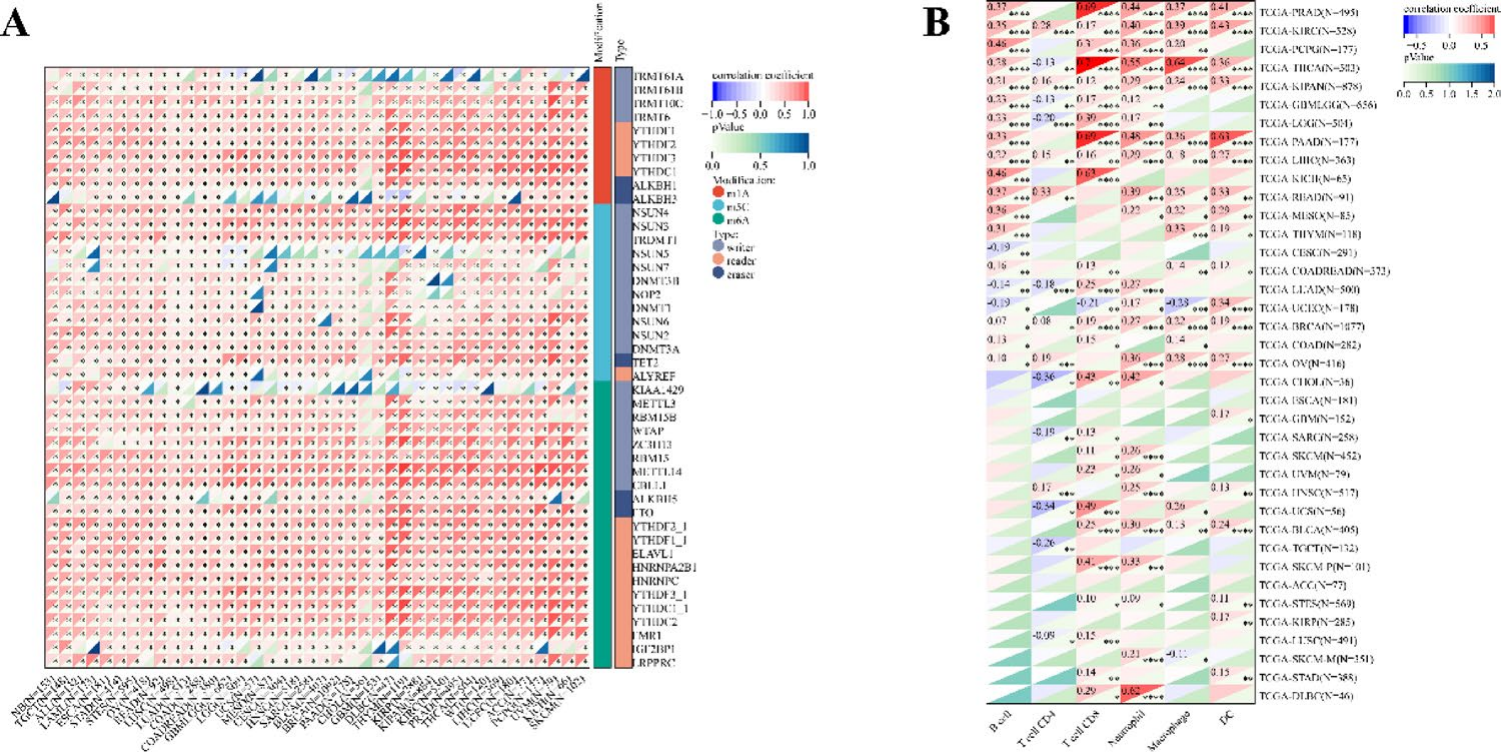
TFAM expression with RNA modification-related genes and immune cell infiltration at pan-cancer level. **A** the correlation between TFAM expression and 44 RNA modification-related genes, including m1A (10), m5C (13) and m6A (21) at pan-cancer level; **B** the correlation between TFAM expression and tumor-infiltrating cells including B cell, T cell CD4, T cell CD8, Neutrophil, Macrophage and DC at pan-cancer level using the TIMER algorithm. *p < 0.05; **p < 0.01; ***p < 0.001; ****p < 0.0001. DC dendritic cell

Using the methods of our previous studies [56, 57], we initially used pan-cancer analysis to explore the association between TFAM and cancers. The differential expression analysis reveals that TFAM is significantly upregulated in 25 tumor tissues compared to normal tissues, including cancers such as Glioblastoma (GBM), Lung Adenocarcinoma (LUAD), and Acute Myeloid Leukemia (LAML) (Fig. 3A). In addition, we observed significant downregulation of TFAM in 5 tumors, including Kidney Papillary Cell Carcinoma (KIRP), Kidney Clear Cell Carcinoma (KIRC), Testicular Cancer (TGCT), Adrenocortical Cancer (ACC) and Kidney Chromophobe (KICH) (Fig. 3A). Mutation profiling highlights that TFAM undergoes various genetic alterations, including nonsynonymous mutations and splice site mutations, particularly in cancers like Cervical Squamous Cell Carcinoma (CESC), Rectal Adenocarcinoma (READ), and Corpus Endometrial Carcinoma (UCEC), suggesting its involvement in tumor-specific molecular mechanisms (Fig. 3B). Survival analyses further underscore the clinical relevance of TFAM (Fig. 3C–E). High TFAM expression is associated with poor prognosis in cancers such as LAML, LUAD, and Pancreatic Adenocarcinoma (PAAD), as indicated by significantly elevated hazard ratios. Conversely, in cancers like KIRC and lower grade glioma and glioblastoma (GBMLGG), elevated TFAM expression correlates with improved patient outcomes, suggesting a context-dependent role of TFAM in cancer biology. Moreover, we analyzed the correlations of TFAM expression with immunomodulatory genes (Fig. 4) and RNA modification-related genes (Fig. 5A) in various tumor contexts. TFAM showed strong positive correlations with several chemokine and immune stimulator genes, suggesting its potential involvement in enhancing immune cell recruitment and activation. Conversely, moderate correlations were observed with immune inhibitors, hinting at a dual regulatory role of TFAM in balancing immune activation and suppression. Additionally, TFAM exhibited strong correlations with key genes responsible for RNA methylation (e.g., METTL3, YTHDF1), RNA editing, and RNA stability. These findings imply that TFAM may influence RNA processing pathways, potentially impacting gene expression regulation in the tumor microenvironment (TME). Lastly, the analysis of TFAM’s role across different cancer types highlights its diverse impact on immune cell infiltration (Fig. 5B). In multiple tumor types from the TCGA dataset, TFAM expression correlated with immune cell infiltration patterns, including T cells, macrophages, and dendritic cells, with cancer-type-specific variation. For instance, in Prostate Adenocarcinoma (PRAD), Thyroid Carcinoma (THCA), PAAD and KICH, TFAM was strongly positively correlated with CD8 + T cells. In Large B-cell Lymphoma (DLBC), TFAM was strongly positively correlated with neutrophils. In THCA, TFAM was strongly positively correlated with macrophages. In PAAD, TFAM was strongly positively correlated with dendritic cells. These results suggest that TFAM may play a complex and important function in the tumor immune microenvironment.

## Role of TFAM in human cancer

### TFAM and digestive system cancers

In 2022, there were 511,000 new cases of pancreatic cancer and 467,000 deaths globally, making the disease the sixth leading cause of cancer mortality among both men and women, accounting for nearly 5% of all cancer-related deaths worldwide. Pancreatic cancer is characterized by an extremely poor prognosis and is regarded as one of the most lethal forms of cancer [58, 59]. TFAM has been unequivocally associated with aggressive tumor biology and poor prognosis in pancreatic ductal adenocarcinoma (PDAC) [60, 61]. Clinically, high TFAM expression, frequently detected in surgical specimens, serves as an independent prognostic marker correlating with advanced tumor stage, metastatic recurrence and significantly shorter patient survival [61]. Mechanistically, it promotes mitochondrial biogenesis and metabolic adaptation, often via upstream regulators like HMGB1 activating the AMPK/SIRT1/PGC-1α axis under hypoxia, thereby fueling cancer cell proliferation and stress resistance [62]. Crucially, TFAM exerts a potent anti-apoptotic effect, partly through regulating survivin expression, which contributes to tumor cell survival [60]. Its involvement in chemotherapy response appears complex yet pivotal; while foundational studies demonstrate that TFAM depletion induces mitochondrial dysfunction and oxidative stress, thereby sensitizing even gemcitabine-resistant cells to treatment, some models suggest acquired resistance may involve metabolic rewiring [63, 64]. Ultimately, this mitochondrial dependency exposes a therapeutic vulnerability, as evidenced by novel agents designed to degrade TFAM (e.g., targeted LONP1 protease), which trigger mtDNA replication blockade and lethal mitophagy [53].

Hepatocellular carcinoma (HCC) is the third most common cause of cancer-related mortality, and the sixth most common type of cancer identified globally. Over 900,000 new cases and over 830,000 deaths from liver cancer are expected to occur globally in 2020. More than 80% of liver cancers are HCC, which are among the top three causes of cancer-related death in 46 countries and among the top five in 90 nations [65–67]. The role of TFAM in HCC exemplifies a complex and context-dependent duality, acting as both a potential facilitator of tumor resilience and a suppressor of metastatic progression. Foundational to mitochondrial biogenesis and function, TFAM is a downstream effector of the NRF1/PGC-1α axis, a pathway itself suppressed by oncogenic factors like ZNF281 [30]. Its knockdown consequently impairs mitochondrial content, oxygen consumption and TCA cycle metabolism, linking mitochondrial integrity to tumor bioenergetics. This pro-mitochondrial function underlies TFAM’s association with chemoresistance, as its upregulation in resistant HCC cells supports survival and nucleoside triphosphate pools, rendering these cells particularly vulnerable to TFAM inhibition [68]. However, contrasting this tumor-supportive role, compelling evidence positions TFAM as a potent metastasis suppressor. TFAM deficiency orchestrates a profound prometastatic program, driving spontaneous lung metastasis in vivo through malonyl-CoA accumulation, mDia2 malonylation and consequent nuclear actin polymerization, which remodels chromatin to activate metastasis-associated genes [69]. Furthermore, TFAM loss facilitates HCC cell invasion by unexpectedly enhancing mycoplasma infection via an Sp1/ANXA2 mechanism and NF-κB pathway activation [20]. This metastasis-suppressive function is clinically relevant, as low TFAM correlates with poor prognosis and tumor recurrence [69]. Thus, TFAM sits at a critical nexus: while its mitochondrial maintenance function can be co-opted to fuel tumor growth and drug resistance, its loss unleashes diverse retrograde signaling cascades that powerfully enable invasion and metastasis, highlighting its dualistic nature and the nuanced consideration required for its potential therapeutic targeting in HCC.

Globally, colorectal cancer (CRC) ranks second in terms of death but third in terms of incidence. Nearly one in ten cancer cases and deaths are predicted to be CRC in 2022, with an estimated 1.9 million new cases and 904,000 fatalities [70, 71]). Within the complex metabolic landscape of CRC, TFAM emerges as a pivotal regulator with a context-dependent and seemingly dualistic role, intricately governing both tumor suppression and promotion. Critically, its function exhibits a stage-specific duality. In the context of normal intestinal epithelium and inflammatory settings (e.g., colitis), loss or impairment of TFAM function compromises cellular homeostasis, thereby increasing the susceptibility to malignant transformation and enhancing the initiation of colitis-associated cancer [19]. Conversely, within already transformed colon cancer cells, experimental knockdown of TFAM significantly diminishes their tumor-initiating potential and tumorigenicity [72]. The underlying mechanism involves a retrograde signaling pathway where TFAM knockdown elevates α-ketoglutarate levels, inhibiting prolyl hydroxylase activity and subsequently downregulating Wnt/β-catenin signaling, a key driver of cancer stemness [72, 73]. Furthermore, low TFAM expression correlates with reduced CD3 + and CD8 + T-cell infiltration in tumors, potentially linking mitochondrial dysfunction to an immunosuppressive microenvironment [74]. Conversely, in established CRC, TFAM frequently assumes an oncogenic role supporting tumor progression. Immunohistochemical analyses reveal that high TFAM expression positively correlates with advanced TNM stage, lymph node metastasis, and poor patient prognosis [75]. Functionally, TFAM overexpression in CRC cells promotes proliferation by enhancing mitochondrial biogenesis and respiratory activity. This aligns with the inverse relationship observed between the chaperone TRAP1 and mitochondrial-encoded proteins; TRAP1 silencing de-represses the PGC-1α/TFAM pathway, boosting mitochondrial biogenesis and facilitating a metabolic shift towards OXPHOS that supports cancer cell growth [76]. The oncogenic function of TFAM in established tumors appears to be driven, in part, by the β-catenin/c-Myc axis, creating a feed-forward loop [19]. An exception to this pattern is found in microsatellite-instable CRCs, where frameshift mutations in *TFAM* cause truncated protein production, mtDNA depletion, and may confer resistance to apoptosis, illustrating how genetic context modifies its role [77]. Collectively, TFAM embodies a metabolic rheostat in CRC: its loss or dysfunction may facilitate tumorigenesis by altering signaling and the TME, while its subsequent upregulation in advanced cancers fuels the bioenergetic and biosynthetic demands of proliferating cells.

In esophageal squamous cell carcinoma (ESCC), the role of TFAM also presents a complex and context-dependent paradigm. Evidence indicates that TFAM reduction disrupts mitochondrial biogenesis, leading to mitochondrial dysfunction, cytosolic mtDNA stress and activation of the cGAS-STING-autophagy axis, which paradoxically promotes ESCC tumor growth [78]. Conversely, other studies demonstrate that a high mtDNA copy number, regulated by TFAM, is associated with enhanced mitochondrial bioenergetics and increased invasiveness through epithelial-mesenchymal transition [79]. Furthermore, a low mtDNA copy number, resulting from TFAM knockdown, is linked to chemotherapy resistance and poor prognosis, potentially mediated through mitochondrial membrane potential depolarization and DNA methylation [80].

In gastric cancer, the role of TFAM is established as a promoter of tumor progression through mechanisms linked to mitochondrial genomic instability and retrograde signaling. Clinically, decreased mtDNA copy number, regulated by TFAM, is a frequent event in gastric adenocarcinomas and is associated with poorer patient survival [81]. Experimental TFAM knockdown recapitulates this phenotype, inducing severe mitochondrial respiratory dysfunction and a compensatory metabolic shift toward glycolysis, which is concomitantly associated with enhanced cancer cell migration. Beyond bioenergetics, TFAM depletion triggers a specific mitochondrial-nuclear retrograde communication pathway. The reduction in mtDNA content, rather than impaired OXPHOS, activates calcium-mediated signaling, leading to the upregulation of genes including CFAP65 and PCK1. This TFAM-mtDNA-calcium-CFAP65-PCK1 axis subsequently alters cancer cell morphology and suppresses proliferation, indicating a complex role in modulating cell fate [82].

**Fig. 6:**
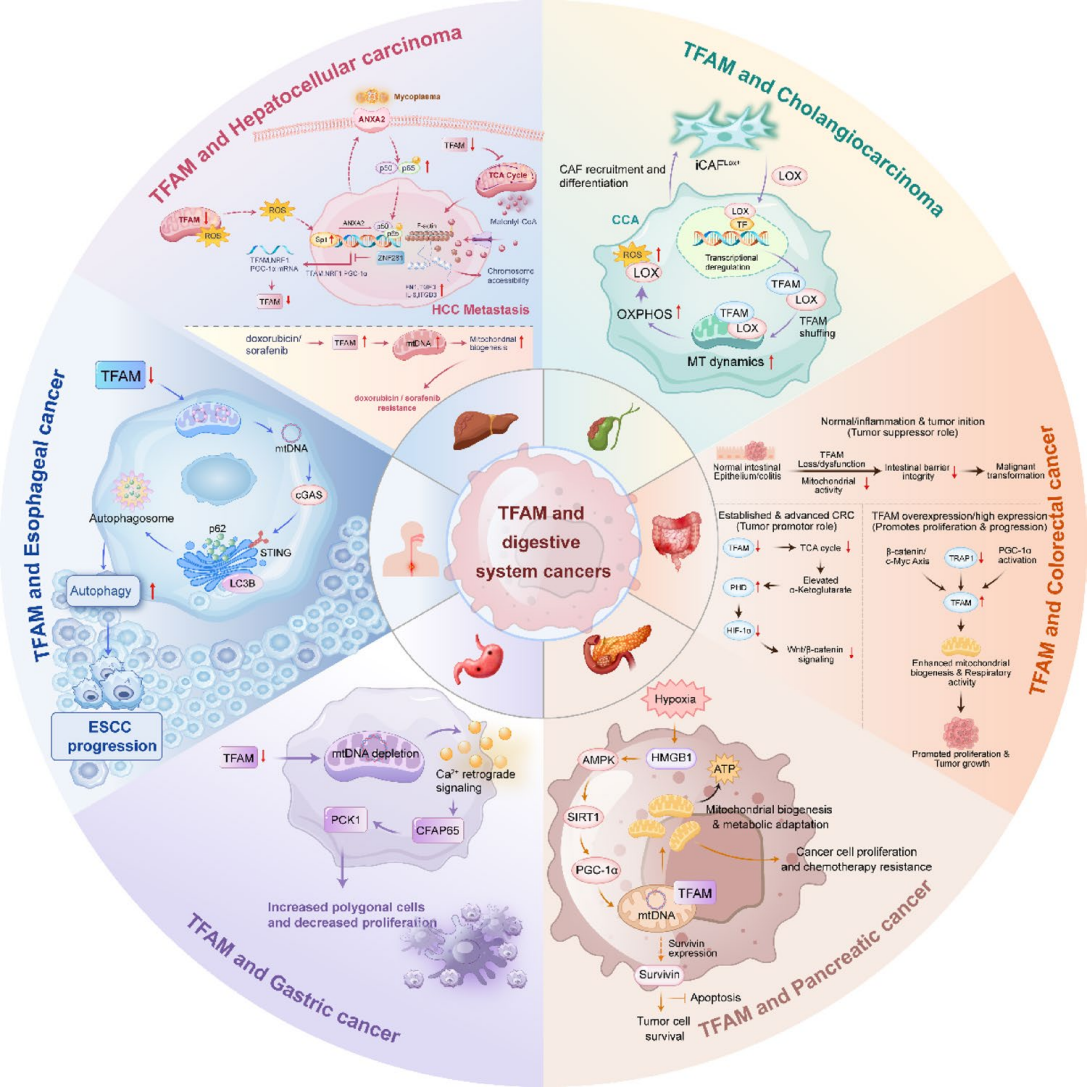
The role of TFAM in digestive cancers. Hepatocellular Carcinoma: TFAM functions as a metastasis suppressor. TFAM deficiency leads to Malonyl-CoA accumulation, inducing mDia2 malonylation and nuclear actin polymerization, which drives chromatin remodeling and metastasis. Additionally, TFAM downregulation facilitates invasion via Sp1/ANXA2-mediated susceptibility to mycoplasma infection and NF-κB activation. Cholangiocarcinoma: CAF-secreted LOX promotes TFAM-dependent mitochondrial function. Colorectal Cancer: TFAM exhibits a dual role. In tumor initiation, TFAM knockdown elevates α-KG, inhibiting PHD to downregulate Wnt/β-catenin signaling. In established tumors, silencing of TRAP1 de-represses the PGC-1α/TFAM axis, boosting OXPHOS to support growth. Pancreatic Cancer: Under hypoxic conditions, HMGB1 activates the AMPK/SIRT1/PGC-1α axis to upregulate TFAM, promoting mitochondrial biogenesis and chemoresistance. Gastric Cancer: TFAM depletion activates (Ca^2^⁺)-dependent retrograde signaling (CFAP65/PCK1) to remodel cell morphology. Esophageal cancer: TFAM reduction disrupts mitochondrial biogenesis, causing cytosolic mtDNA stress that activates the cGAS-STING-autophagy axis, paradoxically promoting tumor growth. ROS reactive oxygen species, TCA tricarboxylic acid, CAF Cancer-associated fibroblast, LOX lysyl oxidase, OXPHOS oxidative phosphorylation, mtDNA mitochondrial DNA

Notably, a recent study [83] focuses on the role of lysyl oxidase (LOX) in cholangiocarcinoma (CCA), revealing that LOX significantly promotes the metabolic fitness and tumor progression of CCA cells by modulating TFAM-mediated mitochondrial function. The research demonstrates that LOX is primarily expressed by cancer-associated fibroblasts (CAFs) and enhances OXPHOS capacity through a TFAM-dependent mechanism, thereby promoting metabolic reprogramming and stemness in CCA cells. Figure 6 depicted the role of TFAM in digestive cancers.

### TFAM and haematological cancers

Hematological malignancies include leukemia, lymphoma, and multiple myeloma, affecting the blood, bone marrow, and lymphatic system. These cancers result from abnormal cell growth or dysfunction, leading to symptoms like anemia, bleeding, and fever. Risk factors include genetics, environmental exposures, and immune system issues [84, 85]. In recent years, mtDNA was found to play a significant role in hematological malignancies by influencing disease development, progression, and therapeutic response through mutations, copy number variations (CNVs), and metabolic regulation [86, 87].

LAML is a fast-growing cancer of the blood and bone marrow characterized by the abnormal proliferation of immature myeloid cells, known as blasts [88, 89]. In LAML, TFAM expression is frequently upregulated, correlating with increased mtDNA copy number and enhanced oxidative metabolism, which independently predicts inferior event-free and overall survival [90]. Mechanistically, TFAM serves as a critical effector downstream of oncogenic signaling pathways, such as the AHR; AHR activation in LAML cells increases TFAM expression, promoting mitochondrial biogenesis, membrane potential and resistance to cytarabine [91]. Conversely, metabolic competition within the TME reveals another facet: leukemic glycolysis can suppress T-cell TFAM, impairing anti-tumor immunity [92]. Thus, TFAM sits at a nexus, where its dysregulation in malignant cells fuels a protumor metabolic state and chemoresistance, while its inhibition in T cells contributes to immune evasion, highlighting its dual role as a potential therapeutic target in LAML.

Chronic Lymphocytic Leukemia (CLL) is a slow-progressing type of blood cancer that originates in the bone marrow and primarily affects B lymphocytes, a type of white blood cell involved in the immune system [93, 94]. Huang et al. [95] found the role of nitric oxide (NO) in driving mitochondrial biogenesis in CLL, with a focus on the key biogenesis factor TFAM. Elevated NO levels in CLL cells correlate with increased mitochondrial mass and enhanced TFAM expression, promoting mitochondrial biogenesis. Furthermore, higher mitochondrial mass, driven by NO and TFAM activity, is linked to reduced sensitivity to the chemotherapeutic drug fludarabine.

Notably, a recent study [96] revealed that mitochondrial transcription and translation, driven by TFAM, were crucial for germinal center B cell function and lymphoma progression. Genetic deletion of *Tfam* in B cells confers protection against lymphoma development in a c-Myc-driven model, highlighting its essential pro-tumorigenic role. Mechanistically, TFAM supports the hyper-proliferative capacity of malignant B cells and maintains cytoskeletal integrity for proper cellular motility and microenvironmental localization. Consequently, pharmacological inhibition of mitochondrial transcription/translation replicates these cytoskeletal defects and potently suppresses the growth of human lymphoma cells.

### TFAM and gynecological cancers

Gynecologic cancers, including endometrial cancer (EC), ovarian cancer (OC) and cervical cancers (CC), are a leading cause of cancer-related deaths among women worldwide [97]. Despite advances in HPV vaccination and screening programs that have reduced cervical cancer incidence, many low-income countries lack large-scale screening, still leading to high mortality rates [98, 99]. In CC, studies consistently demonstrate its overexpression in CC tissues and cell lines compared to normal counterparts, which correlates with advanced clinicopathological parameters and predicts unfavorable overall and disease-free survival [100]. Genetic analyses further implicate *TFAM* in disease phenotype, as specific polymorphisms (e.g., rs3900887) are associated with tumor size [101]. Functionally, TFAM is critical for sustaining malignant behavior. Its downregulation impairs mitochondrial membrane potential and mtDNA copy number while increasing ROS. This mitochondrial dysfunction is coupled with inhibited autophagy processes and reduced expression of metastasis-related proteins. Consequently, suppressing TFAM expression effectively curtails cervical cancer cell proliferation, colony formation, invasion, and migration [102].

EC, the most prevalent gynecological malignancy in Western countries, exhibits a close link between TFAM expression levels and the clinical and pathological features of the tumor, as well as patient prognosis [100, 103]. Higher TFAM levels are linked to more aggressive EC behaviors, including increased cell proliferation, cell cycle advancement, colony formation and cell migration [104–107]. EC is categorized into two main types: estrogen-related (type I, endometrioid) and non-estrogen-related (type II, non-endometrioid) [108]. Cormio et al.’s research indicated significantly higher TFAM levels in type I EC tissues compared to healthy tissues, Mechanistically, this elevation is regulated by PGC-1α, which acts upstream to induce NRF-1, subsequently promoting TFAM expression and mitochondrial biogenesis. This activation of the PGC-1α/NRF-1/TFAM signaling axis leads to elevated protein levels of all three factors and increased citrate synthase activity in mitochondria. Consequently, this results in doubled mitochondrial mass and mtDNA content, which supported the metabolic demands and survival of cancer cells [109]. Tokio et al.’s study substantiated the significant correlation between TFAM expression and various clinical and pathological variables in EC, particularly in type I. It revealed associations between TFAM expression and factors like surgical stage, myometrial invasion, lymphovascular space invasion, cervical invasion, and lymph node metastasis, as well as a positive correlation with p53 expression. Although univariate survival analysis indicated a lower 10-year overall survival rate for patients with TFAM-positive type I EC, TFAM was not identified as an independent prognostic factor in multivariate analysis. These connections were not observed in type II EC patients [110]. EC, particularly type I, is characterized by elevated TFAM expression that correlates with aggressive tumor behavior and poor prognosis, influencing mitochondrial function and potentially serving as a biomarker for clinical outcomes, although its prognostic significance varies by cancer subtype.

In OC, the role of TFAM emerges as multifaceted and context-dependent, integrating mitochondrial integrity, nuclear gene regulation and therapeutic response. TFAM’s expression is frequently altered in ovarian tumors, correlating with distinct clinicopathological features. Studies reveal that TFAM, along with its transcriptional coactivator PGC-1α, exhibits subtype-specific expression patterns; for instance, clear-cell carcinomas (CCC) often show low or absent TFAM/PGC1α, whereas high-grade serous carcinomas (HGSC) frequently display elevated levels [111]. Crucially, this differential TFAM expression does not necessarily reflect total mitochondrial mass, as outer membrane markers like VDAC remain comparable across subtypes. Instead, it signifies a divergence in mitochondrial activity and metabolic wiring: the TFAM-low phenotype in CCC correlates with significant glycogen accumulation, indicating a shift towards glycolysis, while the TFAM-high profile in HGSC supports the intense bioenergetic demands of rapid proliferation [111]. Prognostically, the implications of TFAM are complex. While some immunohistochemical analyses associate high nuclear TFAM expression with poorer survival, potentially via its regulation of anti-apoptotic genes like BCL2L1 in the nucleus [112], other genomic and proteomic data paradoxically indicate that high mitochondrial content and elevated TFAM/TIMM23 expression can be favorable prognostic markers, possibly reflecting a higher sensitivity to platinum-induced apoptosis [113]. Mechanistically, TFAM is central to mitochondrial biogenesis and its upregulation in many ovarian cancers is linked to increased mtDNA content, mitochondrial number, and altered mitochondrial morphology. Critically, mitochondrial biogenesis driven by the PGC1α/TFAM axis enhances mitochondrial ROS production, which appears to be a pivotal determinant of cisplatin sensitivity in HGSC. Reducing TFAM diminishes mitochondrial ROS and confers resistance, whereas increasing mitochondrial content or ROS enhances cisplatin-induced apoptosis [113–115]. Furthermore, somatic mutations in *TFAM* itself have been identified and may co-segregate with therapy resistance [116].

### TFAM and urological cancers

Bladder cancer (BCa) is a prevalent malignancy, yet it has a limited set of reliable prognostic markers and effective molecular targets for therapy [117]. Research indicates that specific Akt isoforms, notably Akt3, are critical for proper mitochondrial respiration, suggesting an isoform-specific upstream regulation of TFAM [118]. Notably, the mechanism by which SQLE stabilizes TFAM protein by interacting with and inhibiting the mitochondrial protease LONP1, thereby driving BCa tumorigenesis, has been elaborated in the preceding “Post-translational modifications and protein stability” section [52]. Prostate cancer (PCa) is the second most frequently observed cancer and the fifth leading cause of cancer deaths among men worldwide [119, 120]. TFAM plays a multifaceted role in prostate carcinogenesis, acting as a critical nexus between environmental insult, mitochondrial dysfunction and malignant progression. Chronic exposure to arsenic, a known carcinogen, promotes cell survival and genotoxicity in human prostate epithelial cells by upregulating TFAM expression via an NRF-1-dependent pathway, thereby altering mitochondrial activity and DNA repair dynamics. This suggests TFAM is a key mediator in arsenic-induced neoplastic transformation [121]. Clinically, TFAM expression is significantly elevated in PCa tissues compared to normal prostate, and its higher levels correlate with poorer patient survival. In vitro functional studies demonstrate that TFAM knockdown inhibits the colony-forming capability of PCa cells, directly implicating it in supporting the proliferative and survival phenotypes of malignancy [122]. Kidney cancer, predominantly renal cell carcinoma (RCC) originating from the renal tubular epithelium, represents a significant global health burden with an estimated 400,000 new cases and nearly 175,000 deaths annually worldwide [123, 124]. In clear cell RCC (ccRCC), suppression of the coactivator PGC-1α downstream of HIF signaling leads to reduced TFAM expression, impairing mitochondrial respiration and promoting a glycolytic shift [125]. Clinically, low TFAM expression is associated with poor patient outcomes, and TFAM is identified within prognostic gene signatures [126]. Experimentally, TFAM knockdown in RCC models decreases mtDNA copy number and oxidative capacity while upregulating glycolytic enzymes, HIF-2α, and oncogenic actors like AKT and MYC, thereby enhancing invasiveness and chemoresistance [127]. Conversely, restoration of mitochondrial function via upstream regulators like SIRT3, which deacetylates and stabilizes TFAM, can reverse the Warburg effect and suppress tumor growth [55].

### TFAM and nervous system cancers

Gliomas are the most common primary tumors of the central nervous system in the adult [128]. GBM is the most frequent and aggressive brain tumor in humans [129]. Various molecules and signaling pathways are involved in its development and progression. TFAM forms nucleoid structures to maintain the integrity of mtDNA. mtDNA depletion has been correlated to cancer progression, and therefore it is considered a potential tool to identify patients with poor prognosis [130–133]. Research had found that in GBM, the overactivation of STAT3 promoted tumor development, and its binding with TFAM could alter mitochondrial function and energy production, leading to tumor progression and resistance to chemotherapy drugs such as temozolomide (TMZ) [134]. Another study found that honatisine, a diterpenoid compound, could reduce TFAM levels, disrupt mtDNA transcription, lead to mitochondrial dysfunction and cell death, effectively reversing the resistance of glioma to TMZ. Animal experiments shown that honatisine had good anti-tumor effects and low toxicity, providing a new strategy for the treatment of GBM and offering a scientific basis for the development of new drugs targeting TFAM [135]. In addition, melatonin reduced the expression of TFAM, TFB1M and TFB2M, disrupted mtDNA transcription, induced an increase in ROS production and mitochondrial damage in tumor cells, and enhanced the efficacy of the chemotherapeutic drug TMZ [136]. In diffusely infiltrating astrocytomas, an increase in POLG expression was associated with a reduction in mtDNA copy number, which in turn correlated negatively with the degree of tumor malignancy. Additionally, TFAM promoted mtDNA replication by activating TFB1M and TFB2M. Although higher TFAM expression might help compensate for the reduction in mtDNA and was related to extended patient survival time, it was still unclear whether it could fully offset the loss or dysfunction of mtDNA [132, 137]. The upregulation of TEFM mediated by TFAM also promotes the progression of low-grade glioma [138]. Studies had demonstrated the crucial role of VEGF and its receptors, VEGFR1 and VEGFR2, in glioma angiogenesis and cell proliferation [139]. Blocking VEGF could reduce tumor vessels, brain oedema, and improved Chemotherapy and radiation outcomes [140, 141]. The expression of VEGFR2 was increased in GBM [142]. Inhibition of VEGFR2 expression activated the AKT-PGC1α-TFAM signaling cascade, increased the expression of mitochondrial proteins and mitochondrial mass, enhanced OXPHOS levels and ROS production, and ultimately promoted tumor cell apoptosis, inhibited tumor growth, arrested the cell cycle and induced cellular senescence [143]. Castracani et al. studied the effects of 17β-estradiol (E2) in GBM. They discovered that E2 in U87-MG cells boosted expressions of mitochondrial health-related genes like PGC1α, SIRT1, TFAM, and also enhanced NRF2 nuclear translocation and heme oxygenase-1 expression. Findings indicate E2’s potential to promote GBM cell proliferation and chemo-sensitivity via mitochondrial and signaling pathway regulation [144].

### TFAM and thoracic cancers

Breast cancer (BRCA) is one of the most common malignancies in female cancers worldwide, with high morbidity and mortality [145]. According to the statistics, more than 2 million women worldwide are diagnosed with BRCA [146] each year. TFAM is upregulated in BRCA tissue and is associated with poor prognosis in BRCA patients [39, 147–149]. In addition, the resistance of estrogen receptor positive BRCA cells to cisplatin chemotherapy is associated with the expression level of TFAM, suggesting that TFAM could be a potential target to overcome chemoresistance [150].

**Fig. 7:**
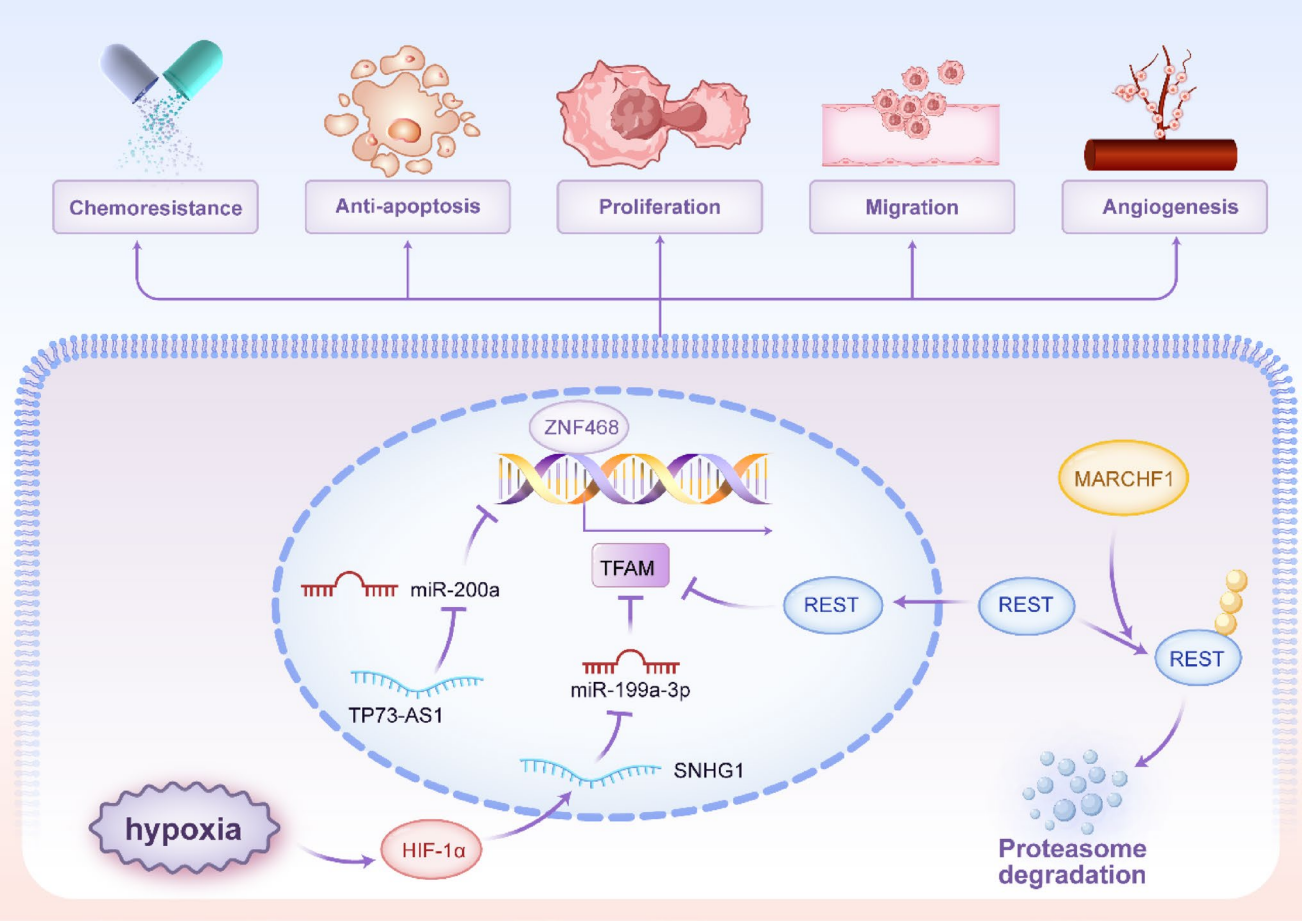
The role of TFAM in breast cancer. Under hypoxia, HIF-1α induces the lncRNA SNHG1, which functions as a competing endogenous RNA to sequester miR-199a-3p, thereby relieving miRNA-mediated repression of TFAM. In parallel, the lncRNA TP73-AS1 sponges miR-200a to alleviate its inhibitory effect on ZNF468, enabling ZNF468-dependent transcriptional activation of TFAM. TFAM expression is additionally constrained by the transcriptional repressor REST; MARCHF1 promotes REST ubiquitination and proteasome-dependent degradation, which attenuates REST-mediated repression and favors TFAM upregulation. Elevated TFAM is linked to enhanced chemoresistance, anti-apoptosis, proliferation, migration and angiogenesis. lncRNA long non-coding RNA, miRNA microRNA, Ub ubiquitination

The expression of TFAM in BRCA is finely regulated by a network of epigenetic and transcriptional mechanisms. As detailed in the “Regulation of TFAM Expression and Activity” section, multiple miRNAs, including miR-200a [39] and miR-199a-3p [46], directly target TFAM to suppress its expression, thereby inhibiting cell proliferation or restoring cisplatin sensitivity. Conversely, the lncRNA TP73-AS1 acts as a ceRNA for miR-200a, leading to TFAM upregulation and promoting BRCA cell proliferation [49]. At the transcriptional level, upstream regulators such as MARCHF1 [32] and ZNF468 [26] enhance TFAM expression, contributing to tumor progression and chemoresistance. Interestingly, VEGF signaling exerts context-dependent effects on mitochondrial biogenesis; while VEGF inhibition can activate the Akt-PGC-1α-TFAM axis and increase mitochondrial biogenesis, elevated VEGF in tumors may suppress this pathway, reflecting the complex, condition-dependent role of TFAM in BRCA [151]. These findings collectively underscore TFAM as a central integrator of diverse regulatory inputs that collectively shape BRCA progression and therapy response. Figure 7 depicted the role of TFAM in BRCA.

Lung cancer is the leading cause of cancer-related deaths globally, accounting for approximately 20% of all cancer mortality [58]. Non-small cell lung cancer (NSCLC) represents about 85% of cases [152, 153]. TFAM plays a critical role in lung tumorigenesis and progression. In oncogenic Kras-driven lung cancer models, loss of TFAM disrupts mitochondrial function and reduces tumorigenesis [154]. In NSCLC cells, TFAM downregulation induces G1 phase arrest, suppresses growth and migration via ROS-mediated JNK/p38 MAPK activation, and reduces cellular bioenergetics, while also enhancing cisplatin-induced apoptosis [155]. Clinically, high TFAM expression correlates with advanced histological grade and TNM stage in NSCLC patients and serves as an independent prognostic factor for poor overall survival [155–157]. Regulation of TFAM expression in NSCLC involves ncRNAs networks. As noted earlier, circ_0002476 is overexpressed in NSCLC and promotes tumor progression by sponging miR-1182, thereby elevating TFAM levels and enhancing mitochondrial function, proliferation, and invasion while reducing apoptosis and mtDNA damage [43]. Additionally, HDAC inhibitors exert therapeutic effects in part by upregulating TFAM expression, restoring mitochondrial function, and modulating immune responses in the TME [158]. These findings highlight TFAM as a metabolic and regulatory node in lung cancer, influencing both tumor cell behavior and therapeutic outcomes.

### TFAM and head and neck cancer

Head and neck cancer (HNC) comprises a group of malignant tumors originating from various anatomical sites in the head and neck region, including the oral cavity, pharynx, larynx, nasal cavity, paranasal sinuses, and salivary glands [159]. Most head and neck cancers are squamous cell carcinomas, closely associated with smoking, excessive alcohol consumption, and human papillomavirus infection [160]. It is estimated that there are approximately 650,000 new cases annually, resulting in over 350,000 deaths worldwide [161]. Li et al. [162] revealed a tumor-suppressive role of TFAM and mtDNA in HNC. TFAM silencing promotes tumor growth, motility, and chemoresistance by reprogramming metabolism toward aerobic glycolysis and activating ERK1/2-Akt-mTORC-S6 signaling. Reduced TFAM and mtDNA levels in HNC tumors correlate with disease progression, suggesting their potential as diagnostic markers and therapeutic targets. In addition, a recent study [163] identified potential prognostic biomarkers for HNC, linking TFAM gene SNPs to patient survival. The TFAM rs11006129 CC genotype and absence of the T allele were associated with longer survival, while the TFAM rs3900887 A allele correlated with shorter survival. These findings suggest that TFAM variants may impair disease outcomes and warrant further investigation in larger, diverse cohorts to validate their prognostic significance. Moreover, in oral squamous cell carcinoma (OSCC), the expression of TFAM is downregulated [164]. Xie et al. [165] identified CKIP-1 as a potential therapeutic target in OSCC. They found that CKIP-1 is highly expressed in OSCC tissues and functions as an oncogene. Mechanistically, CKIP-1 silencing promotes the degradation of TFAM, leading to mitochondrial dysfunction, ROS accumulation and the leakage of mtDNA. This cytosolic mtDNA subsequently activates the cGAS-STING signaling pathway, thereby suppressing tumor growth and inducing apoptosis.

### TFAM and other tumors

Melanoma is a type of skin cancer that originates from melanocytes, the cells responsible for producing melanin, the pigment that gives skin its color [166, 167]. While melanoma primarily develops in the skin, it can also occur in other parts of the body, such as the eyes (ocular melanoma) and mucosal surfaces (e.g., mouth, nasal passages) [168]. Araujo et al. [169] investigated the role of mtDNA and TFAM in melanoma metabolism and tumorigenesis. They revealed a positive correlation between mtDNA copy number, glucose consumption, and ATP production in melanoma cell lines, indicating that TFAM-driven mitochondrial biogenesis supports a high-energy metabolic state utilizing glucose. Conversely, TFAM downregulation resulted in the suppression of glycolytic enzymes and a compensatory shift toward amino acid metabolism. Specifically, TFAM-low cells upregulated glutamine transporters (e.g., SLC1A5) and exhibited growth arrest in glutamine-free conditions, highlighting a dependency on glutamine anaplerosis to sustain survival when glucose metabolism is impaired. In parallel with metabolic reprogramming, Wu et al. [170] uncovered a critical link between TFAM deficiency and therapeutic resistance in melanoma. They demonstrated that partial depletion of TFAM causes mitochondrial stress and the leakage of mtDNA into the cytosol, which triggers the cGAS-STING signaling pathway. Intriguingly, this activation does not induce a canonical inflammatory response but instead upregulates a specific subset of interferon-stimulated genes (ISGs), such as Parp9. These ISGs enhance nuclear DNA repair mechanisms, thereby rendering melanoma cells resistant to DNA-damaging chemotherapeutic agents like doxorubicin. Additionally, TFAM expression is associated with VEGF and promotes an invasive gene expression signature, contributing to tumor progression. Additionally, TFAM has also been found to potentially be associated with uveal melanoma metastasis. A Study showed that uveal metastatic melanoma cell lines exhibit significantly lower mtDNA copy numbers compared to primary cell lines, along with downregulation of mitochondrial biogenesis-related genes such as POLG, TFAM, NRF-1, and SIRT1 [171]. These findings suggest that the patterns of mtDNA variation, copy number, and regulation of mitochondrial biogenesis genes in metastatic cells differ from those in primary cells, providing a potential therapeutic direction for exploring metabolic vulnerabilities associated with uveal melanoma metastasis. Moreover, in terms of gene expression regulation of TFAM, miR-181a/b can reverse melanoma resistance to the BRAF inhibitor dabrafenib by significantly downregulating TFAM expression as mentioned before [172]. In addition, histone variant macroH2A1 can promote the progression of uveal melanoma by regulating mitochondrial function through the modulation of TFAM expression levels [173].

Osteosarcoma is a highly malignant primary bone tumor that primarily originates from osteoblasts, characterized by strong invasiveness and a high propensity for metastasis [174, 175]. It is the most common malignant bone tumor among adolescents and young adults, accounting for the highest proportion of primary bone tumors [176]. Montopoli et al. [177] identified reduced expression of TFAM associated with impaired mitochondrial biogenesis, as a key feature of doxorubicin-resistant osteosarcoma cells. Resistant cells exhibit lower mitochondrial activity, including reduced membrane potential, mass, and ROS production. Notably, combined treatment with doxorubicin and quercetin, a mitochondrial biogenesis inducer, restores sensitivity to doxorubicin, suggesting that targeting TFAM and mitochondrial biogenesis could be a promising strategy to overcome chemotherapy resistance in osteosarcoma. In addition, a study [31] published in *cell metabolism* showed that FBP2, a gluconeogenic isozyme silenced in various soft tissue sarcomas, could suppress tumor growth through dual mechanisms: while cytosolic FBP2 antagonizes the Warburg effect by enzymatically inhibiting glycolysis, nuclear FBP2 exerts a distinct, non-enzymatic function by restraining mitochondrial biogenesis and respiration. Mechanistically, nuclear FBP2 acts as a transcriptional corepressor that physically interacts with c-Myc at the TFAM promoter, thereby blocking c-Myc-driven TFAM transcription and subsequent mitochondrial biogenesis. This unique dual functionality highlights FBP2 as a critical metabolic regulator that coordinates the suppression of both glycolytic and mitochondrial energy pathways in soft tissue sarcomas. Table 1 summarizes the role of TFAM at the pan-cancer level.

**Table 1:** The summary of TMAM at pan-cancer level

Cancer types	Upstream regulators	Expression of upstream regulators	Expression of TFAM in cancer	Clinical Prognostic Association	Target factor	Biological function	Reference (PMID)
Endometrial, ovarian, and cervical cancers	Not applicable	Not applicable	Upregulation	Endometrial: OS HR = 6.34 (*p* = 0.001); DFS HR = 12.97 (*p* < 0.001) Ovarian: OS HR = 2.01 (*p* = 0.003) Cervical: RFS HR = 2.69 (*p* = 0.038)	mtDNA	Related to an unfavorable overall survival and disease-free survival	33042424
Cervical cancer	miRNA-214	Downregulation	Upregulation	Not applicable	mtDNA	Promoted cell proliferation, cell cycle progression, colony-formation, and migration	25556274
Cervical cancer and osteosarcoma	Not applicable	Not applicable	Downregulation	Not applicable	mtDNA	Inhibited autophagy, proliferation, invasion and migration	38710515
High-grade serous ovarian cancer	Not applicable	Not applicable	Downregulation	Not applicable	mtDNA	Attenuated cisplatin induced apoptosis	31699970
Serous ovarian cancer	Not applicable	Not applicable	Upregulation	Poor OS: 5-year survival rate significantly lower in TFAM-positive vs. negative patients (32.0% vs. 43.7%, *p* = 0.021)	BCL2L1	Promoted the survival and proliferation	22098591
Ovarian cancer	Not applicable	Not applicable	Upregulation	Not applicable	mtDNA	Increased mitochondrial number	31547300
Type I endometrial cancer	PGC-1alpha	Upregulation	Upregulation	Not applicable	mtDNA	Increased mitochondrial biogenesis	19861117
Endometrial carcinomas	Not applicable	Not applicable	Upregulation	OS: High expression associated with lower 10-year survival (65.9% vs. 86.4%, *p* = 0.0031)	mtDNA	A maker for progression of the tumors	20232213
Bladder cancer	miRNA-590-3p	Downregulation	Upregulation	Not applicable	mtDNA	Promote cell proliferation, regulate cell cycle, activate PI3K-Akt signaling pathway, and promote tumor invasion and metastasis	24137381
Clear cell renal cell carcinoma	PGC-1α	Downregulation	Downregulation	Not applicable	mtDNA	Impaired mitochondrial respiration	26119730
Renal cell carcinoma	Not applicable	Not applicable	Downregulation	Not applicable	mtDNA	Conferred renal cell carcinoma more invasive and a drug-resistant phenotype	27231905
Clear cell renal cell carcinoma	SIRT3	Upregulation	Upregulation	Not applicable	mtDNA	Promoted mitochondrial biogenesis	30095923
Low grade glioma	Not applicable	Not applicable	Upregulation	Positively correlates with malignancy (WHO grade). Highly co-expressed with TEFM, which is an independent risk factor (OS HR > 1, *p* < 0.01)	mtDNA	Predicted poor overall survival	35117724
Glioblastoma	estradiol	Upregulation	Upregulation	Not applicable	mtDNA	Promoted cell proliferation and mitochondrial fitness	32148493
Glioblastoma	STAT3	Upregulation	Not applicable	Not applicable	mtDNA	TFAM inhibition suppressing mtDNA transcription and inducing lethal respiratory dysfunction	32165236
Glioblastoma	mitochondria	Upregulation	Downregulation	Not applicable	mtDNA	Disrupted mtDNA expression	29747444
Glioblastoma	VEGFR2	Downregulation	Upregulation	Not applicable	mtDNA	Enhanced mitochondrial biogenesis	38702818
Glioma	miRNA-23b	Downregulation	Upregulation	Not applicable	mtDNA	Enhanced cell proliferation, migration, invasion	24002170
Glioma	Not applicable	Not applicable	Upregulation	REMBRANDT cohort indicating a strong association with clinical prognosis and tumor development	Not applicable	Be a diagnostic marker	28440425
Glioblastoma	honatisine	Upregulation	Downregulation	Not applicable	mtDNA	Induced apoptosis	38522316
Pancreatic cancer	LONP1	Upregulation	Downregulation	Not applicable	POLG, PINK1–Parkin axis	Suppressed pancreatic cancer proliferation	37969736
Pancreatic cancer	NRF-1	Upregulation	Upregulation	Not applicable	mtDNA	Enhanced mitochondrial biogenesis	32103987
Pancreatic cancer	Not applicable	Not applicable	Upregulation	Not applicable	mtDNA	Enhanced mitochondrial biogenesis	37002014
Pancreatic ductal adenocarcinoma	Not applicable	Not applicable	Downregulation	Not applicable	mtDNA	Increased resistance to gemcitabine	35887170
Pancreatic cancer	PGC-1α	Downregulation	Downregulation	Not applicable	mtDNA	Reduced mitochondrial biogenesis	31629659
Hepatocellular carcinoma	Not applicable	Not applicable	Upregulation	Not applicable	mtDNA	Correlated with tumor size	29137431
Hepatoma	NRF-1	Upregulation	Upregulation	Not applicable	mtDNA	Increased mtDNA content	16230352
Hepatocellular carcinoma	Not applicable	Not applicable	Upregulation	Not applicable	mtDNA	Were more sensitive to TFAM inhibition	32461017
Hepatocellular carcinoma	p53	Downregulation	Downregulation	Not applicable	mtDNA	Inhibited mitochondrial biogenesis and functions	24801417
Liver cancer	Not applicable	Not applicable	Downregulation	TFAM downregulation is significantly associated with shorter OS and time to recurrence (*p* < 0.05)	mtDNA	Promoted metastasis	35451091
Hepatocellular carcinoma	ZNF218	Upregulation	Downregulation	Not applicable	mtDNA	Reduced mitochondrial biogenesis	37880213
Colorectal cancer	p53	Not applicable	Upregulation	High expression is an independent predictor of poor 5-Year Survival (*p* = 0.003) and significantly correlated with advanced TNM stage (*p* = 0.003) and lymph node metastasis	mtDNA	Increased mitochondrial copy number	27732955
Colorectal cancer	Not applicable	Not applicable	Downregulation	Not applicable	mtDNA	Induced mitochondrial DNA depletion and apoptotic resistance	21467167
Colon cancer	PGC-1α, Nrf2, HO-1, AMPK, mTOR and p53	Upregulation	Upregulation	Not applicable	mtDNA, COX-IV	Prevented colon cancer growth	28986187
Colon cancer	miR-204	Upregulation	Downregulation	Not applicable	Not applicable	Up-regulated the proliferation	26499153
Colorectal cancer	miR-214	Downregulation	Upregulation	Not applicable	Not applicable	Decreased proliferation	30157478
Colorectal cancer	mitochondrial calcium uniporter	Upregulation	Upregulation	Not applicable	mtDNA	Enhanced mitochondrial biogenesis	32371956
Colorectal cancer	PKA	Downregulation	Upregulation	Not applicable	mtDNA	Enhanced growth	34235078
Colorectal cancer	PGC-1α, NRF1	Upregulation	Downregulation	Not applicable	mtDNA	Induced mitochondrial dysfunction	36591497
Colon cancer	miR-590-3p	Downregulation	Downregulation	Not applicable	mtDNA	Inhibited cancer growth	27878255
Colorectal cancers	PGC-1α	Upregulation	Upregulation	Not applicable	mtDNA	Enhanced mitochondrial biogenesis	39210159
Colorectal cancer	Not applicable	Not applicable	Upregulation	Not applicable	mtDNA	Increased mitochondrial function	29138850
Esophageal squamous cell carcinoma	Not applicable	Not applicable	Upregulation	Not applicable	mtDNA	Increased invasiveness	23109849
Esophageal squamous cell carcinoma	Not applicable	Not applicable	Downregulation	Low TFAM expression significantly correlates with shorter OS (*p* < 0.05) and enhanced tumor growth	mtDNA	Promoted cancer growth	35750756
Acute myeloid leukemia	Not applicable	Not applicable	Downregulation	Not applicable	mtDNA	Decreased mitochondrial biogenesis	39457563
B cell chronic lymphocytic leukemia	nitric oxide	Upregulation	Upregulation	Not applicable	mtDNA	Enhanced mitochondrial biogenesis	15483672
Breast cancer	Not applicable	Not applicable	Upregulation	Not applicable	mtDNA	Contributed to cisplatin resistance	27779689
Breast cancer	miR-200a	Upregulation	Downregulation	Not applicable	mtDNA	Attenuated cell proliferation	24684598
Breast cancer	MiR-199a-3p	Upregulation	Downregulation	Not applicable	mtDNA	Enhanced sensitivity to cisplatin	28126676
Breast cancer	HIF-1, SNHG1, miR-199a-3p	Upregulation	Upregulation	Not applicable	mtDNA	Promoted breast cancer development and metastasis	34003544
Breast cancer	TP73-AS1	Upregulation	Downregulation	Not applicable	mtDNA	Promoted breast cancer cell proliferation	28639399
Breast cancer	Membrane associated ring-CH-type finger 1	Upregulation	Upregulation	Not applicable	mtDNA	Promoted breast cancer progression	39428668
Breast cancer	zinc finger protein 468	Upregulation	Upregulation	Not applicable	mtDNA	Promoted breast cancer cell growth and migration	38429817
Breast cancer	PGC1α	Downregulation	Upregulation	Not applicable	mtDNA	Enhanced mitochondrial biogenesis	33628590
Lung cancer	Not applicable	Not applicable	Downregulation	Not applicable	mtDNA	Reduced tumorigenesis	20421486
Non-small cell lung cancer	Not applicable	Not applicable	Downregulation	OS: RR = 2.272 (*p* = 0.017)	JNK/p38MAPK	Reduced cellular bioenergetics	26820294
Non-small cell lung cancer	circ_0002476	Upregulation	Upregulation	Not applicable	mtDNA	Promoted progression	36056804
Non-small cell lung cancer	NF-κB/HIF1α axis	Upregulation	Upregulation	Not applicable	mtDNA	Recovery of dysfunctional mitochondria	37473712
Head and neck cancer	Not applicable	Not applicable	Downregulation	TFAM Mutation (K141N): Associated with significantly shorter median survival (22.19 vs 56.94 months, *p* < 0.05)	mtDNA	Decreased tumour-suppressing role	34663785
Oral squamous cell carcinoma	CKIP-1	Downregulation	Downregulation	Not applicable	mtDNA	Suppressed cancer	39169452
Uveal melanoma	Not applicable	Not applicable	Downregulation	Not applicable	mtDNA	Promoted metastatic	34082186
Melanoma	miR-181 mimics	Upregulation	Downregulation	Not applicable	mtDNA	Inhibited melanoma growth	33670365
Uveal melanoma	macroH2A1	Downregulation	Downregulation	Not applicable	mtDNA	Reduced the aggressiveness	32401230

### Therapeutic strategies targeting TFAM

Despite the compelling evidence positioning TFAM as a central hub in cancer metabolism and signaling, targeting TFAM clinically remains a formidable challenge. Historically considered “undruggable” due to its lack of deep hydrophobic pockets and its essential role in normal physiology [178], recent advances have begun to unravel potential avenues for therapeutic intervention. We categorize current strategies into direct modulation, indirect pathway regulation, and novel delivery approaches.

### Direct modulation by small molecules and natural products

Given the structural challenges, natural products and drug repurposing have emerged as effective strategies. Honatisine, a diterpenoid alkaloid, overcomes TMZ resistance in GBM by inducing mitochondrial unfolded protein responses to disrupt TFAM function [135]. In HCC, molecular docking reveals that Oroxylin A directly binds TFAM, promoting p53-mediated degradation and inhibiting protective mitophagy to reverse sorafenib resistance [179]. Drug repurposing offers a rapid translational path: the antiviral Zalcitabine promotes TFAM degradation and ferroptosis, a distinct type of programmed cell death driven by iron-dependent oxidative stress [180], in pancreatic cancer [181], while the OXPHOS inhibitor Atovaquone triggers endoplasmic reticulum stress in ovarian cancer. Crucially, Atovaquone induces the extracellular release of TFAM as a Damage-Associated Molecular Pattern (DAMP), thereby priming the TME for NK cell-mediated immunity [182].

Conversely, in contexts where restoring mitochondrial function is therapeutic, Quercetin acts as a TFAM inducer to re-establish chemotherapy sensitivity in osteosarcoma [177]. Similarly, Kaempferol exerts anti-colorectal cancer effects through multi-target metabolic remodeling; by targeting TFAM to enhance OXPHOS while simultaneously inhibiting glycolytic enzymes, it triggers a lethal surge in ROS and mitochondrial potential collapse [183].

### Indirect regulation via metabolic and signaling pathways

Targeting the regulatory machinery of TFAM offers an alternative strategy. Transcriptional repression is effective in metastatic models: Agrimol B and Silibinin inhibit the PGC-1α/NRF axis to suppress TFAM expression and epithelial-mesenchymal transition in colon and breast cancers, respectively [21, 22]. Expanding on this, Dynorphin A upregulates SP-1 to suppress the PGC-1α/Nrf1/TFAM axis, impairing bioenergetics in osteosarcoma [184]. Specific metabolic vulnerabilities can also be exploited, such as Terbinafine targeting the SQLE-LONP1 axis in BCa, releasing Lon protease to degrade TFAM [52].

### Precision delivery and genetic tools

To address the risk of off-target toxicity in high-energy organs (heart/muscle), mitochondria-targeted nanocarriers represent a major breakthrough [185]. Miriplatin-loaded liposomes have been engineered to degrade TFAM and POLG specifically in pancreatic cancer mitochondria [53]. More recently, Metal–Organic Frameworks (MOFs) like Res@ZIF-90 have utilized the acidic TME for pH-responsive release. This platform delivers resveratrol directly to gastric cancer mitochondria, disrupting TFAM homeostasis and inhibiting growth more effectively than free drugs [186]. Table 2 summarizes the potential therapeutic strategies targeting TFAM.

**Table 2:** Summary of potential therapeutic strategies targeting TFAM

Category	Agent/tool	Mechanism of action	Target cancer/context	Reference (PMID)
Inhibitors/downregulators	Honatisine	Induces mitochondrial UPR; disrupts TFAM function	Glioblastoma (TMZ resistance)	38522316
	Melatonin	Downregulates TFAM, TFB1M, TFB2M; induces ROS	Glioblastoma	29747444
	Zalcitabine	Promotes TFAM degradation; induces ferroptosis	Pancreatic cancer	32186434
	Atovaquone	Inhibits OXPHOS; induces ER stress and extracellular release of TFAM as DAMP	Epithelial ovarian cancer	41286323
	Oroxylin A	Binds TFAM; promotes p53 degradation to inhibit mitophagy	Hepatocellular carcinoma (Sorafenib resistance)	39770569
Stabilizers/inducers	Quercetin	Induces mitochondrial biogenesis and TFAM expression	Osteosarcoma	36900165
	Tetramethylpyrazine	Inhibits Lon protease to stabilize TFAM	General/Cytoprotection	28465355
	Kaempferol	Targets TFAM to enhance OXPHOS/ROS; inhibits TKT and ALDOA	Colorectal cancer	41292411
	17β-estradiol (E2)	Upregulates PGC-1α, SIRT1, and TFAM	Glioblastoma	32148493
Indirect regulators (metabolic/signaling)	Agrimol B	Inhibits PGC-1α/NRF1/TFAM axis	Colon cancer	36591497
	Terbinafine	Inhibits SQLE to restore Lon-mediated TFAM degradation	Bladder cancer	41254141
	Silibinin	Downregulates PGC-1α/NRF2/TFAM axis; promotes mitochondrial fusion	Triple-negative breast cancer (Metastasis)	31612353
	Ethyl p-methoxycinnamate	Inhibits H-ras/c-Myc signaling to downregulate TFAM	Ehrlich ascites tumor	37408910
	Dynorphin A	Upregulates SP-1 to suppress PGC-1α/NRF1/TFAM axis	Osteosarcoma	40836639
Genetic/delivery-based approaches	Miriplatin-loaded liposomes (LMPt)	Mitochondria-targeted degradation of TFAM and POLG	Pancreatic cancer (Gem resistance)	37969736
	Res ZIF-90 (MOF)	pH-responsive mitochondrial targeting; downregulates PGC-1α/TFAM	Gastric cancer	39509747

## Discussion and perspective

### Context-dependent roles of TFAM in tumorigenesis

The multifaceted involvement of TFAM in cancer progression presents an apparent paradox: it functions as an essential oncogene in certain malignancies while exhibiting tumor-suppressive characteristics in others. This duality is not contradictory but rather reflects the tissue-specific metabolic flexibility and signaling thresholds inherent to different TME. First, in tumors characterized by high metabolic demands and reliance on OXPHOS, such as PDAC and CRC, TFAM acts primarily as a bioenergetic driver [19, 63, 64, 73]. In these contexts, TFAM upregulation is required to maintain mitochondrial biogenesis and ATP production, thereby supporting rapid proliferation. Consequently, TFAM inhibition in these “metabolically addicted” tumors precipitates an energy crisis, leading to growth arrest and chemosensitization. Second, in malignancies like melanoma or head and neck squamous cell carcinoma, TFAM downregulation acts as a trigger for distinct signaling cascades. Reduced TFAM levels compromise the structural integrity of the mitochondrial nucleoid, leading to the leakage of mtDNA into the cytosol. This event activates the cGAS-STING pathway [165, 170]. While typically associated with immune surveillance, chronic low-level activation of this pathway in the TME can paradoxically drive metastasis, promote epithelial-mesenchymal transition, and induce an immunosuppressive phenotype, thus mimicking a “tumor-suppressive” loss but resulting in aggressive outcomes [187, 188]. Finally, the conflicting findings regarding drug resistance, such as the fact that TFAM downregulation can confer either sensitivity or resistance, can be reconciled by the “TFAM threshold effect”. Severe depletion of TFAM can induce lethal mitochondrial dysfunction and trigger ROS-mediated apoptosis, thereby sensitizing cancer cells. In contrast, moderate suppression may simply reduce basal ROS levels, enabling cells to escape oxidative stress-induced death. It may also activate compensatory survival mechanisms, for example through the upregulation of DNA repair genes via interferon signaling. Thus, whether TFAM acts as a friend or foe hinges on a delicate balance: the tumor’s metabolic reliance on mitochondria and the specific downstream signaling pathways activated under mitochondrial stress.

### Bridging transcriptomic profiling and biological reality

A critical comparison between our pan-cancer RNA-sequencing analysis and experimental literature reveals both striking alignments and instructive divergences. In metabolically rigid tumors like KIRC, bulk transcriptomic data perfectly mirrors experimental findings where TFAM loss drives a pseudo-hypoxic, glycolytic switch promoting aggression. However, high-throughput bulk sequencing can obscure dynamic, stage-specific mechanisms [189]. For instance, while our analysis indicates general TFAM upregulation in HCC, experimental models delineate a metastatic switch where TFAM must be specifically downregulated to unleash nuclear actin-mediated invasion [69]. This discrepancy suggests that bulk RNA sequencing captures the proliferative signal of the primary tumor core but misses the transient, low-TFAM states of metastatic sub-clones. Similarly, while our correlation analysis links TFAM expression to high CD8 + T-cell infiltration, experimental evidence clarifies that TFAM is also crucial for the maintenance of immunosuppressive regulatory T cells (Tregs). Thus, the “hot” tumor phenotype inferred from bulk immune deconvolution might functionally represent a Treg-dominated, immune-excluded microenvironment.

To resolve these ambiguities, emerging single-cell RNA sequencing (scRNA-seq) technologies offer a transformative solution by unmasking intra-tumor heterogeneity [190]. Unlike bulk profiling, single-cell analysis can distinguish between a TFAM-high proliferative cluster and a TFAM-low invasive subpopulation within the same tumor mass, thereby reconciling the paradox of its dual roles [189]. Furthermore, high-resolution immune profiling can precisely pinpoint TFAM expression levels within specific T-cell exhaustion subsets or macrophage polarization states, which bulk-level analysis often conflates. Future studies integrating pseudotime trajectory analysis will be particularly valuable, allowing researchers to trace the dynamic fluctuation of TFAM during cancer progression and determine whether TFAM downregulation is a permanent loss or a reversible state facilitating metastasis.

### Orchestrating metabolic plasticity: TFAM at the crossroads of tumor adaptation

The traditional dichotomy between the Warburg effect and OXPHOS is increasingly being replaced by a systems-level understanding of metabolic plasticity, the dynamic ability of cancer cells to rewire their bioenergetic networks to survive microenvironmental stress and therapeutic insults [191–193]. In this evolving paradigm, TFAM emerges not merely as a supporter of respiration, but as a critical “rheostat” that governs the mitochondrial capacity required for this adaptive switching.

A key aspect of this plasticity is the “hybrid” metabolic phenotype, where aggressive tumor cells simultaneously utilize glycolysis and OXPHOS [194]. High TFAM expression is pivotal for maintaining the mitochondrial arm of this hybrid state, allowing cells to efficiently switch energy sources when glucose is scarce or when glycolytic pathways are targeted. This flexibility is particularly evident during the metastatic cascade. While primary tumor growth often favors glycolysis (“Grow”), the detachment and migration phases (“Go”) require efficient ATP production and resistance to anoikis, processes heavily dependent on PGC-1α/TFAM-mediated mitochondrial biogenesis [195]. Consequently, TFAM enables the metabolic flexibility required for circulating tumor cells to survive the hostile bloodstream and colonize distant organs.

Furthermore, TFAM-driven plasticity plays a central role in therapeutic resilience. Chemotherapy and targeted agents often select for slow-cycling “persister” cells that shift their metabolism toward OXPHOS to evade treatment [196]. In contexts like melanoma and leukemia, this metabolic shift is underpinned by elevated TFAM levels, which maintain mitochondrial integrity despite cytotoxic stress. From a systems biology perspective, TFAM acts as an integration node, translating upstream stress signals (e.g., nutrient deprivation, drug pressure) into a tangible mitochondrial output. Therefore, future therapeutic strategies should look beyond simply inhibiting TFAM to induce cell death; the goal should be to target TFAM to “lock” cancer cells into a rigid metabolic state, stripping them of the plasticity needed to evolve and resist therapy. Breaking this mitochondrial flexibility could be the key to overcoming multi-drug resistance in heterogeneous tumors.

### TFAM modulation of tumor immunity and the microenvironment

While increasing evidence delineates TFAM’s pivotal role in regulating cancer cell proliferation, metabolic reprogramming, and drug resistance, the precise mechanisms by which it orchestrates the complex TME remain a critical frontier for exploration. The TME is a dynamic and complex ecosystem consisting of tumor cells, various host cells such as immune cells, fibroblasts and endothelial cells, along with the extracellular matrix and signaling molecules [197–199]. Beyond its intrinsic functions within tumor cells, TFAM acts as a master regulator of the metabolic and immunogenic crosstalk between malignant cells and the stromal ecosystem, including tumor-associated macrophages (TAMs), dendritic cells (DCs), fibroblasts and infiltrating lymphocytes. A comprehensive analysis of TFAM at the pan-cancer level suggests that its influence extends beyond intracellular bioenergetics; it actively shapes immune escape mechanisms and therapeutic responses by modulating the immunometabolic landscape of the TME.

One of the most significant mechanisms linking TFAM to innate immunity is the regulation of mtDNA integrity and the cGAS-STING signaling axis. A recent study demonstrated that the specific deletion of TFAM in dendritic cells induces severe mitochondrial dysfunction, leading to the cytosolic leakage of mtDNA. This aberrant accumulation of cytosolic DNA triggers the activation of the cGAS-STING pathway, resulting in the robust production of type I interferons and enhanced antigen presentation [200]. Crucially, these changes can reverse the immunosuppressive nature of the TME, effectively turning “cold” tumors “hot” and inhibiting tumor growth and metastasis.

Furthermore, TFAM dictates the metabolic phenotype of stromal cells, creating a supportive niche for tumor progression. Research by Balliet et al. [201] demonstrated that inducing mitochondrial dysfunction in fibroblasts via TFAM knockdown is sufficient to synthetically generate a CAFs phenotype. These TFAM-deficient fibroblasts undergo metabolic reprogramming toward aerobic glycolysis, characterized by the overproduction of hydrogen peroxide (oxidative stress) and the enhanced secretion of L-lactate. This secreted lactate serves as a high-energy fuel that drives mitochondrial OXPHOS in adjacent cancer epithelial cells, a phenomenon termed the “Reverse Warburg Effect”. In xenograft models, these glycolytic fibroblasts significantly promoted tumor growth while exhibiting a loss of Caveolin-1, a stromal biomarker associated with poor clinical prognosis. These findings suggest that TFAM deficiency-mediated oxidative stress mimics “accelerated host aging” within the TME, creating a permissive metabolic niche that sustains malignant progression.

In the adaptive immune compartment, TFAM-mediated mitochondrial respiration is indispensable for the maintenance and function of Tregs. TFAM deficiency impairs Treg stability and homing capabilities by disrupting the OXPHOS necessary for Foxp3 expression. While this leads to tissue inflammation, it concurrently enhances anti-tumor immunity by alleviating Treg-mediated suppression [202]. Adding to this complexity, TFAM functions as a DAMP when released into the extracellular space during cell apoptosis. Extracellular TFAM interacts with the AGER/RAGE, triggering pro-inflammatory signaling that aids in immune surveillance and the clearance of tumor cells [203].

**Fig. 8:**
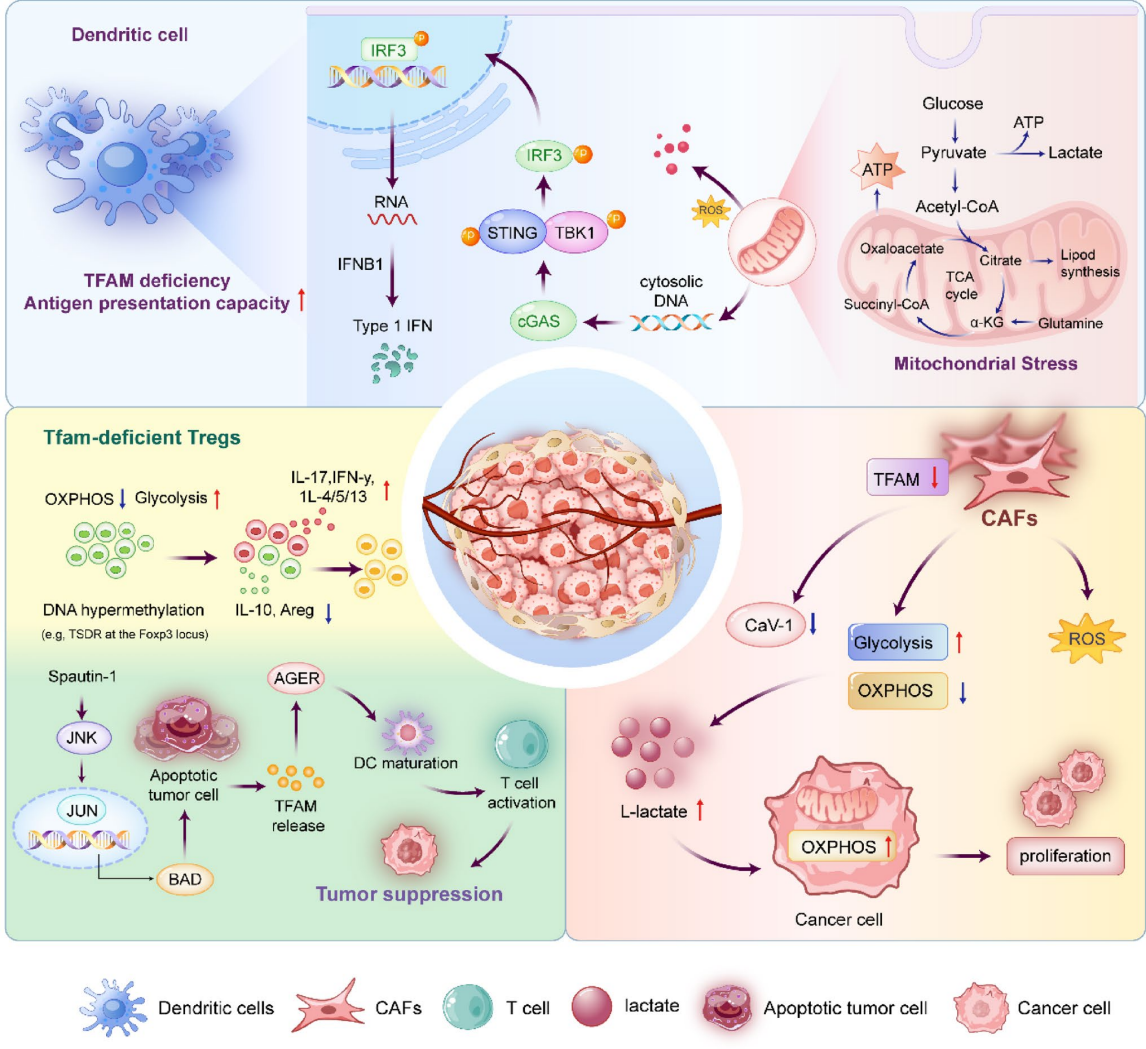
The role of TFAM in tumor microenvironment. In DC, TFAM deficiency enhances antigen presentation capacity. Mitochondrial stress increases ROS and promotes leakage/accumulation of cytosolic DNA, activating the cGAS-STING-TBK1 axis and downstream IRF3 phosphorylation, which induces IFNB1 and type I IFN responses. In Tregs, TFAM-deficient display reduced OXPHOS and increased glycolysis, epigenetic alterations (e.g., DNA hypermethylation at the *Foxp3* locus TSDR) and a cytokine shift characterized by increased IL-17/IFN-γ and type 2 cytokines (IL-4/5/13) with reduced IL-10 and Areg, collectively favoring anti-tumor immunity. In addition, TFAM release from apoptotic tumor cells can promote DC maturation via AGER, leading to T-cell activation and tumor suppression; the Spautin-1-JNK-JUN-BAD pathway is shown as one route enhancing tumor-cell apoptosis and TFAM release. In CAFs, TFAM downregulation decreases CaV-1, increases glycolysis, reduces OXPHOS and elevates ROS, resulting in increased L-lactate export that fuels cancer cell mitochondrial metabolism and proliferation. DC dendritic cell, ROS reactive oxygen species, Tregs regulatory T cells, OXPHOS oxidative phosphorylation, CAFs Cancer-associated fibroblasts, TCA tricarboxylic acid

Crucially, the interaction between TFAM and immune checkpoints represents a vital area of therapeutic relevance. Mitochondrial stress and ROS accumulation, often resulting from aberrant TFAM activity, can stabilize HIF-1α [204]. This transcription factor directly upregulates PD-L1 on tumor cells, establishing an immunosuppressive barrier [205]. Furthermore, while the TFAM-loss-induced cGAS-STING activation promotes T-cell infiltration, chronic interferon signaling can paradoxically induce feedback upregulation of PD-L1, leading to adaptive immune resistance [206]. This duality suggests that targeting TFAM could be a “double-edged sword” for immunotherapy. Simply inhibiting TFAM might activate anti-tumor immunity via cGAS-STING but could simultaneously fuel tumor growth via stromal lactate or induce checkpoint expression. Therefore, a promising precision oncology strategy would be combining TFAM-targeting agents with immune checkpoint inhibitors. For instance, TFAM inhibition could “prime” the tumor by enhancing T-cell infiltration, while anti-PD-L1 antibodies would counteract the potential compensatory checkpoint upregulation. Future studies should focus on defining the “TFAM threshold” that maximizes immunogenicity while minimizing metabolic support for the tumor, paving the way for rational combination therapies. Figure 8 depicted the complex role of TFAM in the TME.

### Challenges and future perspectives in TFAM-targeted therapy

Although the identification of TFAM modulating agents marks a significant leap forward, the transition from preclinical models to clinical oncology faces substantial hurdles. The primary bottleneck lies in the therapeutic window and off-target toxicity. Since TFAM is indispensable for mtDNA maintenance in healthy tissues with high energy demand such as the myocardium, skeletal muscle, and central nervous system, systemic TFAM inhibition poses a high risk of inducing severe mitochondrial cytopathies. Therefore, the development of tumor homing delivery systems is not merely an enhancement but a necessity. Future efforts must prioritize next generation nanocarriers decorated with tumor specific ligands like folate and RGD peptides or pH sensitive motifs that restrict drug release strictly to the acidic TME to minimize systemic exposure [207].

Furthermore, the context dependent duality of TFAM mandates a paradigm shift from universal inhibition to precision patient stratification. As discussed previously, treating a tumor where TFAM downregulation drives metastasis, exemplified by specific subtypes of HCC or melanoma, with a TFAM inhibitor could disastrously accelerate disease progression via retrograde signaling. Consequently, clinical trials must incorporate robust predictive biomarkers like the ratio of mtDNA to nuclear DNA or real time metabolic phenotyping to accurately segregate patients who will benefit from TFAM suppression from those requiring TFAM restoration [208, 209].

Finally, the frontier of TFAM targeting lies in leveraging next-generation pharmacology to overcome its historical “undruggability”. Since traditional small-molecule inhibition is hindered by the lack of deep binding pockets, future efforts should pivot toward advanced modalities that govern protein stability and biophysical integrity. Emerging technologies such as Proteolysis Targeting Chimeras (PROTACs) and molecular glues [210, 211] provide a groundbreaking solution to degrade TFAM directly [212]. By engineering molecules that recruit the cellular ubiquitin–proteasome system, researchers can induce the specific elimination of TFAM, bypassing the requirement for active site occupancy. Furthermore, targeting the mitochondrial transcriptional machinery offers a novel therapeutic modality to functionally suppress mitochondrial gene expression and impair tumor growth [213]. Integrating these cutting-edge chemical biology approaches with systems immunology will define the next era of mitochondrial oncology, transforming TFAM from a challenging target into a potent effector of precision medicine.

## Conclusion

TFAM emerges as a pivotal regulator in cancer biology, exhibiting paradoxical roles as both an oncogenic driver and a tumor suppressor depending on the tissue context. It orchestrates tumor progression not only through metabolic reprogramming and mitochondrial biogenesis but also by shaping the immune microenvironment. While targeting TFAM holds significant therapeutic promise, challenges regarding tissue specificity and drug delivery remain. Future precision oncology strategies must focus on unraveling its dynamic regulatory mechanisms and developing novel delivery systems or combination therapies to effectively balance metabolic inhibition with immune activation.

## Data Availability

No datasets were generated or analysed during the current study.

## Abbreviations

TFAM: The Transcription Factor A, Mitochondrial
mtDNA: Mitochondrial DNA
ROS: Reactive oxygen species
OXPHOS: Oxidative phosphorylation
ncRNAs: Non-coding RNAs
miRNAs: MicroRNAs
lncRNAs: Long non-coding RNAs
circRNAs: Circular RNAs
ceRNA: Competing endogenous RNA
PTMs: Post-translational modifications
GBM: Glioblastoma
LUAD: Lung adenocarcinoma
LAML: Acute myeloid leukemia
KIRP: Kidney papillary cell carcinoma
KIRC: Kidney clear cell carcinoma
TGCT: Testicular cancer
ACC: Adrenocortical cancer
KICH: Kidney chromophobe
CESC: Cervical squamous cell carcinoma
READ: Rectal adenocarcinoma
UCEC: Corpus endometrial carcinoma
PAAD: Pancreatic adenocarcinoma
GBMLGG: Lower grade glioma and glioblastoma
TME: Tumor microenvironment
PRAD: Prostate adenocarcinoma
THCA: Thyroid carcinoma
DLBC: Large B-cell lymphoma
PDAC: Pancreatic ductal adenocarcinoma
HCC: Hepatocellular carcinoma
CRC: Colorectal cancer
ESCC: Esophageal squamous cell carcinoma
GC: Gastric cancer
LOX: Lysyl oxidase
CCA: Cholangiocarcinoma
CAFs: Cancer-associated fibroblasts
CNVs: Copy number variations
CLL: Chronic lymphocytic leukemia
NO: Nitric oxide
EC: Endometrial cancer
OC: Ovarian caner
CC: Cervical cancers
CCC: Clear-cell carcinomas
HGSC: High-grade serous carcinomas
BCa: Bladder cancer
PCa: Prostate cancer
RCC: Renal cell carcinoma
ccRCC: Clear cell renal cell carcinoma
TMZ: Temozolomide
E2: 17β-Estradiol
BRCA: Breast cancer
NSCLC: Non-small cell lung cancer
HNC: Head and neck cancer
OSCC: Oral squamous cell carcinoma
ISGs: Interferon-stimulated genes
DAMP: Damage-associated molecular pattern
MOFs: Metal–organic frameworks
Tregs: Regulatory T cells
scRNA-seq: Single-cell RNA sequencing
TAMs: Tumor-associated macrophages
DCs: Dendritic cells
PROTACs: Proteolysis targeting chimeras
